# Wet Mechanochemical Synthesis of BH_4_‐Substituted Lithium Argyrodites

**DOI:** 10.1002/smtd.202401046

**Published:** 2024-09-05

**Authors:** Ji‐Hoon Han, Yoonju Shin, Young Joo Lee, Sangdoo Ahn, Young‐Su Lee, Kyung‐Woo Yi, Young Whan Cho

**Affiliations:** ^1^ Energy Materials Research Center Korea Institute of Science and Technology (KIST) Seoul 02792 Republic of Korea; ^2^ Department of Materials Science and Engineering Seoul National University Seoul 08826 Republic of Korea; ^3^ Metropolitan Seoul Center Korea Basic Science Institute (KBSI) Seoul 03759 Republic of Korea; ^4^ Department of Chemistry Chung‐Ang University Seoul 06974 Republic of Korea

**Keywords:** all‐solid‐state batteries, argyrodite, lithium borohydride, solid electrolyte, wet mechanochemical synthesis

## Abstract

In all‐solid‐state batteries, a solid electrolyte with high ionic conductivity is required for fast charging, uniform lithium deposition, and increased cathode capacity. Lithium argyrodite with BH_4_^−^ substitution has promising potential due to its higher ionic conductivity compared to argyrodites substituted with halides. In this study, Li_5.25_PS_4.25_(BH_4_)_1.75_, characterized by a high ionic conductivity of 13.8 mS cm^−1^ at 25 °C, is synthesized via wet ball‐milling employing o‐xylene. The investigation focused on optimizing wet ball‐milling parameters such as ball size, xylene content, drying temperature, as well as the amount of BH_4_^−^ substitution in argyrodite. An all‐solid‐state battery prepared using Li_5.25_PS_4.25_(BH_4_)_1.75_ as the electrolyte and LiNbO_3_ coated NCM811 as the cathode exhibits an initial coulombic efficiency of 90.2% and maintains 93.9% of its initial capacity after 100 cycles at fast charging rate (5C). It is anticipated that the application of this wet mechanochemical synthesis will contribute further to the commercialization of all‐solid‐state batteries using BH_4_‐substituted argyrodites.

## Graphical abstract

A method for the synthesis of BH_4_‐substituted argyrodite‐type solid electrolytes via a wet mechanochemical process is established. They have high ionic conductivity of up to 13.8 mS cm^−1^ and the ionic conductivity is strongly dependent on the drying temperature. This wet processing is expected to enable the mass production of fine and uniform powder of highly conducting BH_4_‐substituted argyrodite electrolytes.

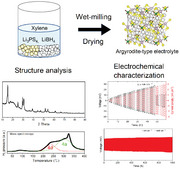

## 1 Introduction

Amidst the expansion of the electric vehicle market and growing interest in next‐generation batteries, all‐solid‐state batteries (ASSBs) are gaining attention as a safer and higher energy‐density alternative to lithium‐ion batteries.^[^ ^1^ ^]^ The substitution of flammable and volatile liquid electrolytes with non‐flammable solid electrolytes enhances safety, and the application of Li metal to the anode or even anode‐less electrode design enables the fabrication of high‐capacity batteries with suppressed Li dendrite formation.^[^ ^2^ ^]^ Lithium argyrodite, a type of lithium thiophosphate, is one of the promising candidates for commercial ASSB due to its high ionic conductivity and good formability.^[^ ^3^ ^]^ Argyrodite electrolytes, which have a cubic structure such as Li_6_PS_5_X (X = Cl, Br, I), were first reported by Deiseroth et al., in 2008.^[^ ^4^ ^]^ By substituting S^2−^ ions at the 4a and 4d Wyckoff sites with X^−^ anions, it is possible to significantly enhance the ionic conductivity.^[^ ^3^, ^5^ ^]^

Research on substituting S^2−^ with BH_4_^−^ in argyrodite has been limited, due to the challenges associated with synthesis, making it less explored. In 2013, Yamauchi et al. first synthesized BH_4_‐substituted argyrodite with the composition (100−x)(0.75Li_2_S‐0.25P_2_S_5_)+xLiBH_4_, demonstrating an ionic conductivity of 1.8 mS cm^−1^ at 25 °C.^[^ ^6^ ^]^ Dao et al.,^[^ ^7^ ^]^ and Wang et al.,^[^ ^8^ ^]^ synthesized partially BH_4_‐substituted Li_6_PS_5_Cl, reporting low ionic conductivities of 0.4 and 0.12 mS cm^−1^, respectively. In our previous study,^[^ ^9^ ^]^ we demonstrated the decomposition of BH_4_‐substituted argyrodite when heat treated at high temperatures. Furthermore, we illustrated the impracticality of Li_2_S‐P_2_S_5_‐LiBH_4_ system due to the irreversible reaction between LiBH_4_ and P_2_S_5_. Hence, the low ionic conductivities observed in previous studies appear to be a result of milling all precursors simultaneously and the high heating temperatures employed during the synthesis. Indeed, Sun et al.,^[^ ^10^ ^]^ recently synthesized Li_5.91_PS_4.91_(BH_4_)_1.09_ without heat treatment and obtained a much improved ionic conductivity of 4.8 mS cm^−1^ at 25 °C. Furthermore, by ball‐milling LiBH_4_ and Li_3_PS_4_, we synthesized Li_6−a_PS_5−a_(BH_4_)_1+a_ with high ionic conductivities of up to 11 mS cm^−1^ at 25 °C.^[^ ^11^ ^]^ In the subsequent study, BH_4_^−^ and X (X = Cl^−^, Br^−^, I^−^) were co‐substituted into S^2−^ sites in the argyrodite structure through ball‐milling, resulting in electrolytes with a high ionic conductivity of up to 16.4 mS cm^−1^ at 25 °C.^[^ ^9^ ^]^

As of now, most Li argyrodite electrolytes are synthesized through high‐energy ball‐milling (HEBM) followed by heat treatment on a laboratory scale. However, HEBM is not easy to be applied for mass production while maintaining controlled particle size and shape. In contrast, the wet chemical process has advantages in terms of mass production, lower process cost, and finer particle size with a relatively narrow size distribution.^[^ ^3^, ^12^ ^]^ However, the wet chemical process presents challenges such as by‐product formation, the generation of electroconductive materials through co‐crystallization from the nucleophilic attack of the solvent, and the need for extended processing times for thorough solvent removal.^[^ ^13^ ^]^ So far, BH_4_‐substituted argyrodites have been synthesized primarily by a dry ball‐milling method, and studies on wet chemical synthesis have not been reported as far as we know. In this study, we synthesized Li‐argyrodites with the composition Li_6−a_PS_5−a_(BH_4_)_1+a_ by partially substituting S^2−^ of the non‐bridging sulfur sites in argyrodite with BH_4_^−^ through a wet mechanochemical process. The synthesized electrolytes exhibited a maximum conductivity of 13.8 mS cm^−1^ at room temperature after vacuum drying. To achieve high ionic conductivity, various process parameters, such as ball size, solvent amount, drying temperature, as well as the amount of BH_4_^−^ substitution in the argyrodite structure, were optimized. The structure and the site occupancies of the anionic species were analyzed by Rietveld refinement of the powder X‐ray diffraction (XRD) data. Furthermore, the presence of residual solvent after vacuum drying was checked via thermogravimetric analysis‐mass spectrometry (TGA‐MS) technique, as well as solid‐state nuclear magnetic resonance (NMR) spectroscopy.

## 2 Results and Discussion

### 2.1 Optimization of the Liquid Phase Ball‐Milling Condition and Chemical Composition

As described in a previous study,^[^ ^9^ ^]^ LiBH_4_ undergoes an irreversible and highly exothermic reaction with P_2_S_5_, necessitating milling with pre‐synthesized Li_3_PS_4_ to form an argyrodite phase. While β‐Li_3_PS_4_ can exhibit an ionic conductivity of ≈1 mS cm^−1^ when synthesized via appropriate dry ball‐milling,^[^ ^9^ ^]^ several studies suggest a decrease of approximately one order of magnitude in ionic conductivity when synthesized through wet chemical methods.^[^ ^12^, ^14^ ^]^ Therefore, β‐Li_3_PS_4_ was synthesized by dry ball‐milling a mixture of Li_2_S and P_2_S_5_. Mixtures of β‐Li_3_PS_4_ and LiBH_4_ were wet ball‐milled using o‐xylene as a solvent. O‐xylene was chosen because it does not react with Li_3_PS_4_ or LiBH_4_. Other xylene isomers and toluene can also be used as the solvent. The synthesized samples were dried by vacuum pumping for 1 hour at room temperature to remove the solvent and subsequently analyzed by XRD. Prior to electrochemical impedance spectroscopy (EIS) measurements, any residual solvent was further eliminated by heating under a dynamic vacuum at 90 °C for 2 hours.

**Figure 1:**
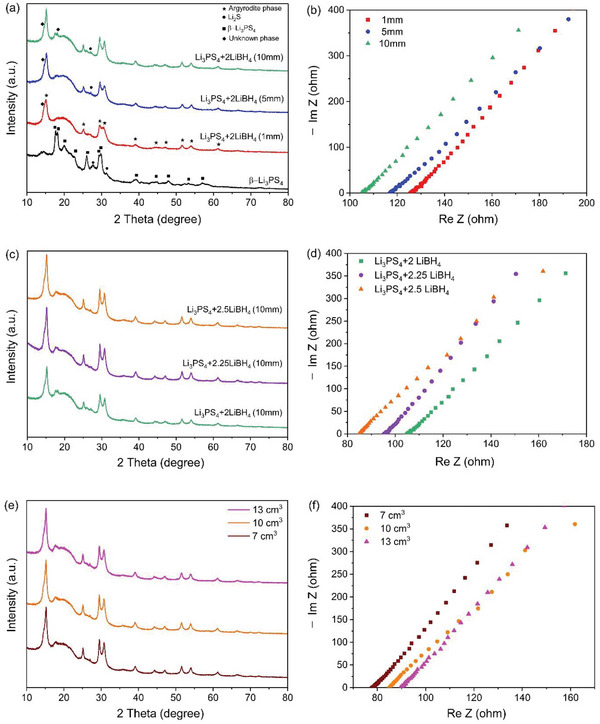
a,b) XRD and EIS data of β‐Li_3_PS_4,_ Li_3_PS_4_+2LiBH_4_ with different ball sizes. c,d) XRD and EIS data for Li_3_PS_4_+xLiBH_4_ synthesized with 10 mm balls through wet ball milling (x = 2, 2.25, 2.5). e,f) XRD and EIS data for Li_3_PS_4_+2.5LiBH_4_ with varying solvent amounts, using 10 mm balls.

First, the influence of ball size on the formation of argyrodite and ionic conductivity was investigated. The synthesis must be accomplished solely through ball‐milling around room temperature due to the thermal decomposition of BH_4_‐substituted argyrodite during heat treatment at high temperatures.^[^ ^9^ ^]^ However, when the ball size is too small, insufficient milling energy leads to the formation of second phase(s), thereby lowering the conductivity.^[^ ^11^ ^]^ When using the PULVERISETTE 7, considering the internal volume of the milling container, using balls larger than 10 mm results in very few balls being used. Additionally, with the same total weight, the number of balls decreases, reducing the collision frequency and thus lowering the reactivity. Therefore, a maximum 10 mm ball was used. **Figure** **1**a shows the XRD data of the Li_3_PS_4_+2LiBH_4_ samples synthesized via wet ball‐milling with different ball sizes using the same total weight of balls (100 g). As the ball size increased, the intensity of the diffraction peaks from the argyrodite phase increased, while the proportion of the unknown phase decreased. Accordingly, the ionic conductivity measured by EIS increased with the increase in ball size (Figure 1b). This can be attributed to the formation of other thiophosphates, containing P_2_S_7_^4−^ or P_2_S_6_^4−^, known to reduce ionic conductivity when smaller ball sizes were employed.^[^ ^11^ ^]^ The samples synthesized with 1 and 5 mm balls exhibited weaker peak intensity from the argyrodite phase and the presence of an unknown phase, making Rietveld refinement almost impossible. In contrast, the electrolyte synthesized with 10 mm balls generates a diffraction pattern with better quality allowing further refinement. Table S2 (Supporting Information) indicates that BH_4_^−^ is substituted at the 4a and 4d sites of the argyrodite, with respective substitution amounts of 0.92 and 0.54. Consequently, the electrolyte has a composition of Li_5.54_PS_4.54_(BH_4_)_1.46_. Therefore, the 10 mm balls were selected for subsequent syntheses due to their optimal crystallinity and high ionic conductivity. In wet ball milling, the particle size of the synthesized powder can vary depending on the size of the balls used. This particle size can influence the ionic conductivity when the powder is pelletized. However, due to the relatively soft mechanical properties of BH_4_‐substituted argyrodite compared to conventional argyrodite, the particle size has minimal impact.

Second, to investigate the effect of the amount of LiBH_4_ addition on the degree of substitution, synthesis was conducted with a varying amount of LiBH_4_ (2, 2.25, 2.5 moles) per one mole of Li_3_PS_4_. When producing BH_4_‐substituted argyrodite through HEBM, excess LiBH_4_ is required because some of it decomposes during ball milling.^[^ ^9^, ^10^ ^]^ Figure 1c displays the XRD data with increasing amounts of LiBH_4_. Due to the larger size of BH_4_^−^ compared to S^2−^, the diffraction peaks shift to lower angles with the increasing addition of BH_4_^−^. An increase in ionic conductivity was observed with the increase of LiBH_4_ as shown in Figure 1d. The Rietveld refinement results for the electrolyte with 2.25 LiBH_4_ showed that BH_4_^−^ was substituted at the 4a and 4d sites of argyrodite with occupancies of 1.0 and 0.67, respectively, indicating the composition of Li_5.33_PS_4.33_(BH_4_)_1.67_ (Table S3, Supporting Information). In the case of adding 2.5 LiBH_4_, the composition becomes Li_5.25_PS_4.25_(BH_4_)_1.75_. (see Table S4, Supporting Information). With the increase in the amount of BH_4_^−^ substitution, both ionic conductivity and lattice parameter increased (Figure S1, Supporting Information), and we therefore chose Li_3_PS_4_+2.5 LiBH_4_ as the optimal composition.

Third, the amount of o‐xylene addition was varied for wet ball‐milling of Li_3_PS_4_+2.5 LiBH_4_ in order to change the slurry viscosity (see **Table** **1**). Solid loading refers to the amount of solid material in a solution or slurry, which is related to the viscosity of the mixture. The viscosity of the slurry in wet ball milling is crucial as it may significantly control the progress of the reaction. If too much solvent is used, the viscosity of the slurry will be too low and most of the milling energy will be used to move the fluid, leaving insufficient energy for the mechanochemical reaction. Conversely, if too little solvent is used, the viscosity of the slurry becomes too high, which can negate the advantages of continuous processes in wet synthesis. Figure 1e depicts the XRD of Li_5.25_PS_4.25_(BH_4_)_1.75_ samples with different amounts of added o‐xylene. No significant difference, in terms of the amount of BH_4_^−^ substitution and lattice parameter, was found from the Rietveld refinement result for the three datasets (Figure S2; Tables S4–S6, Supporting Information). However, as indicated in Figure 1f, the ionic conductivity increased with decreasing amount of solvent addition. Ionic conductivity improved as less solvent was used; however, if less than 7 cm^3^ of solvent was used, the viscosity of the slurry would become too high to be adopted for the wet milling process. Therefore, it is important to maintain an appropriate slurry viscosity that can promote both high ionic conductivity and facile continuous wet milling processing.

**Table 1:** Ionic conductivity of various electrolytes synthesized in different conditions (amount of solvent, ball size, and drying temperature).

Electrolyte	Amount of solvent [cm^3^]	Solid loading [wt%]	Ball size [mm]	Drying temperature [°C]	Ionic conductivity at 25 °C [mS cm^−1^]
Li_3_PS_4_ + 2LiBH_4_	10	20.3	1	90	8.6
Li_3_PS_4_ +2LiBH_4_	10	20.3	5	90	9.2
Li_3_PS_4_ + 2LiBH_4_	10	20.3	10	90	10.1
Li_3_PS_4_ + 2.25LiBH_4_	10	20.7	10	90	11.7
Li_3_PS_4_ + 2.5LiBH_4_	7	27.7	10	90	13.8
Li_3_PS_4_ + 2.5LiBH_4_	10	21.1	10	90	13.2
Li_3_PS_4_ + 2.5LiBH_4_	13	17.1	10	90	12.0
Li_3_PS_4_ + 2.5LiBH_4_	10	21.1	10	50	10.7
Li_3_PS_4_ + 2.5LiBH_4_	10	21.1	10	70	11.5
Li_3_PS_4_ + 2.5LiBH_4_	10	21.1	10	110	12.0
Li_3_PS_4_ + 2.5LiBH_4_	10	21.1	10	130	8.2
Li_3_PS_4_ + 2.5LiBH_4_	10	21.1	10	150	2.9
Li_3_PS_4_ + 2.5LiBH_4_	−	−	10	−	12.9
Li_3_PS_4_ + 2.5LiBH_4_	−	−	10	25	12.5
Li_3_PS_4_ + 2.5LiBH_4_	−	−	10	90	15.1
Li_3_PS_4_ + 2.5LiBH_4_	−	−	10	110	14.6
					

Lastly, the temperature dependence of the ionic conductivity of the electrolyte with the highest ionic conductivity, namely the combination of Li_3_PS_4_+2.5 LiBH_4_, 10 mm ball, and 7 cm^3^ of solvent, was measured. The activation energy *E_a_* of the electrolyte was calculated according to the Arrhenius equation:

(1) $$\sigma \;T\;=\;A\exp\left(-\frac{E_{a}}{RT}\right)$$

where T is the absolute temperature, A is a pre‐exponential factor, and R is the universal gas constant. As shown in Figure S3 (Supporting Information), the activation energy was ≈40.3 kJ mol^−1^.

### 2.2 Optimization of Drying Temperature for Ionic Conductivity

**Figure 2:**
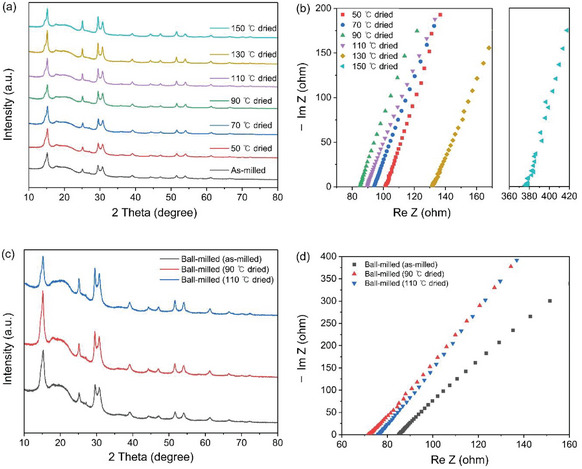
a,b) XRD and EIS measurement data of Li_3_PS_4_ + 2.5LiBH_4_ synthesized via wet ball‐milling at various drying temperatures. c,d) XRD and EIS measurement data of Li_3_PS_4_ + 2.5LiBH_4_ synthesized through dry ball‐milling at different heating temperatures.

In the previous section, the drying temperature was fixed to 90 °C, and other parameters were optimized. Here, we focus on finding an optimal drying temperature that maximizes the ionic conductivity of Li_3_PS_4_ + 2.5LiBH_4_ samples synthesized with 10 mm balls and 10 cm^3^ o‐xylene. **Figure** **2**a shows the XRD data with different drying temperatures under a dynamic vacuum for 2 h. It is observed that the peak intensity generally increases with increasing drying temperature (Figure S4a, Supporting Information). This is because the size of the crystals increased with increasing temperature (Figure S4b, Supporting Information). In contrast to the monotonic increase in the XRD peak intensity with drying temperature, the ionic conductivity reached a maximum value at 90 °C and decreased with increasing temperatures (see Figure 2b). More prominent conductivity decrease above 110 °C is most likely due to the thermal decomposition of BH_4_‐substituted argyrodite at high temperatures;^[^ ^9^ ^]^ in the present study, it starts to decrease at comparatively lower temperature due to the dynamic vacuum condition.

To confirm that the decrease in the ionic conductivity above 110 °C is mainly due to the thermal decomposition and not directly related to the solvent, the sample with the same composition was prepared by dry ball‐milling under the same milling condition, followed by vacuum drying at 90 and 110 °C, respectively. Similar to the case when o‐xylene was used, there was hardly any change in the diffraction patterns, except a slight increase in the peak intensity due to the growth of crystallite size and/or of crystallinity, as shown in Figure 2c. Additionally, as shown in Figure 2d, the dry ball‐milled samples also exhibited the highest ionic conductivity after drying at 90 °C, and a relatively lower value at 110 °C. Thus, it can be concluded that BH_4_‐substituted argyrodite undergoes thermal decomposition at temperatures above ≈100 °C under dynamic vacuum, regardless of the use of solvent.

**Figure 3:**
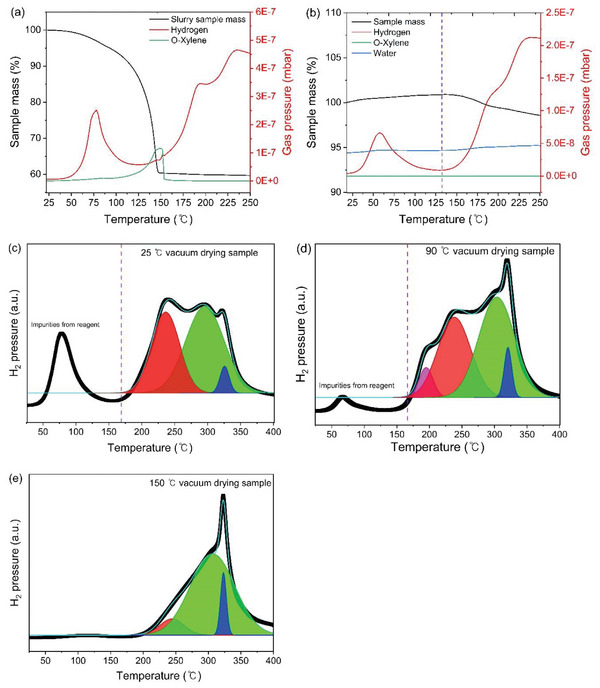
a) TGA‐MS data for slurry of Li_3_PS_4_ + 2.5LiBH_4_ synthesized via wet ball‐milling. b) TGA‐MS data for Li_3_PS_4_ + 2.5LiBH_4_ synthesized via wet ball‐milling after drying at 25 °C for 1 h. c–e) TGA‐MS data measured from 25 to 400 °C of samples dried at 25, 90, and 150 °C, respectively.

To examine the decomposition temperature, the extent of decomposition, and the decomposition products, thermogravimetric analysis coupled with mass spectrometry (TGA‐MS) was conducted. **Figure** **3**a shows the TGA‐MS data of the as‐milled slurry sample containing solvent that was not dried. O‐xylene gradually increased and reached a peak ≈150 °C followed by a sudden drop to the baseline. Hydrogen was detected below 100 °C, which is confirmed to have originated from impurities in the pristine LiBH_4_, as shown in Figure S5a (Supporting Information). Figure S6a (Supporting Information) shows the XRD data for these impurities. Due to its high reactivity with water, LiBH_4_ contains impurities such as HBO_2_ and H_3_BO_3_, and a small amount of LiCl, which has been used in the production of LiBH_4_. Additionally, in previous research, ^11^B NMR confirmed the presence of borates such as BO_3_ and BO_4_ in the LiBH_4_ reagent.^[^ ^9^ ^]^ To prove that the hydrogen peak observed below 100 °C did not originate from the argyrodite, we synthesized the electrolyte by ball‐milling using LiBD_4_ as the reagent and then measured both hydrogen and deuterium using mass spectrometry. As shown in Figure S6b (Supporting Information), while a hydrogen peak was detected below 100 °C, the deuterium peak was detected at much lower levels, indicating that the peak did not originate from the electrolyte. Figure 3b shows the TGA‐MS data of the sample dried at 25 °C. At temperatures above 150 °C, the electrolyte underwent a two‐step decomposition, leading to the production of hydrogen. While these two‐stage peaks were absent in the pristine LiBH_4_, they were evident in all other BH_4_‐substituted argyrodite samples including the dry ball‐milled electrolyte (Figure S5b, Supporting Information). This suggests that the hydrogen generation at temperatures above 150 °C is linked to the thermal decomposition of argyrodite containing BH_4_^−^ at both 4a and 4d sites. Rietveld refinement analysis of samples exposed to different drying temperatures shows that BH_4_^−^ at 4d site start to decrease at a lower temperature compared to BH_4_^−^ at 4a site (Figure S7a, Supporting Information). Hence, it can be inferred that the first H_2_ peak originates from BH_4_^−^ at 4d site, while the subsequent peak originates from BH_4_^−^ at 4a site. Based on the above interpretation, hydrogen MS curves were deconvoluted as shown in Figure 3c–e and S5c–g (Supporting Information). It shows a similar trend to the substitution ratio obtained from Rietveld refinement (see Figure S7, Supporting Information; **Table** **2**). This phenomenon was also observed in the sample synthesized by dry ball‐milling, where a clear mass loss and detection of H_2_ began at temperatures above 150 °C (see Figure S5b, Supporting Information). Drying under 70 bar of hydrogen at 120 °C could not suppress the thermal decomposition as shown in Figure S8 (Supporting Information). When BH_4_^−^ decreases, S^2−^ must substitute for BH_4_^−^ at the 4a and 4d sites within the argyrodite structure. Additionally, since BH_4_^−^ is a monovalent anion and S^2−^ is divalent, an increase in Li^+^ is also necessary to maintain charge balance. These Li^+^ and S^2−^ can be supplied from decomposed argyrodite. In summary, the decrease in ionic conductivity with increasing drying temperature above 100 °C is primarily due to the reduction in the amount of BH_4_^−^ substitution at the 4a and 4d sites in argyrodite, as well as the decrease in the phase fraction of the argyrodite itself.

**Table 2:** Proportion (in %) of BH_4_^−^ located at the 4a and 4d sites extracted from XRD and TGA‐MS profiles (also shown in Figure S6).

Drying temperature [°C]	Proportion from XRD [Rietveld refinement]		Proportion from TGA‐MS [Deconvolution]	
	4a	4d	4a	4d
25	52	48	59	41
50	57	43	65	35
70	57	43	61	39
90	57	43	58	42
110	66	34	80	20
130	74	25	75	25
150	88	12	90	10

The ionic conductivity increases with increasing drying temperature up to ≈100 °C. There are several factors that could have influenced the conductivity. First, the amount of residual o‐xylene would decrease with increasing temperature and the ionic conductivity would increase accordingly. However, it has been confirmed by TGA‐MS analysis that o‐xylene was almost eliminated simply by drying at room temperature for 1 h. Moreover, the same trend in conductivity (see Figure 2b) was also observed in samples synthesized by dry ball‐milling. Second, the distribution of BH_4_^−^ at 4a and 4d sites may change depending on drying temperature, resulting in a change in ionic conductivity. It is well recognized that higher substitution of non‐bridging S^2−^ anions and a more disordered distribution between 4a and 4d sites lead to enhanced ionic conductivity.^[^ ^15^ ^]^ However, the distribution of BH_4_^−^ at 4a and 4d sites does not appear to vary significantly below 90 °C, as shown in Figure S7 (Supporting Information) and Table 2. Therefore, it seems improbable that the improvement in ionic conductivity would be attributed to changes in the total amount of substitution or the distribution over 4a and 4d sites. Third, the impurities present in the pristine LiBH_4_ might have decreased the ionic conductivity. The hydrogen peak observed between 50 and 75 °C (Figures 3 and S5, Supporting Information), which originates from the impurities in pristine LiBH_4_, diminishes as the drying temperature rises. Lastly, the change of microstructures after annealing, such as increased crystallite size, would have increased the ionic conductivity. As shown in Figure S4b (Supporting Information), the crystallite size starts to increase above 50 °C and saturated at above 150 °C. This indicates that the decrease in interfacial resistance caused by the increase in crystallite size may induce the increase in ionic conductivity up to the decomposition temperature of ≈100 °C.^[^ ^16^ ^]^ In conclusion, it is concluded that the enhancement of ionic conductivity is attributed to grain growth, recovery, and recrystallization, as well as the removal of impurities rather than by the removal of residual solvent.

### 2.3 Effect of Drying Temperatures on the Structure

**Figure 4:**
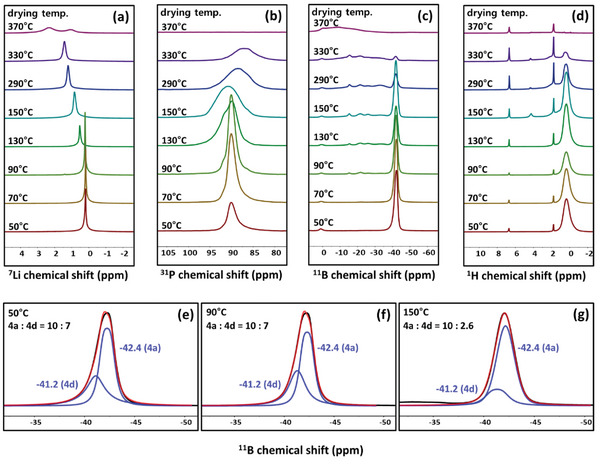
a) ^7^Li, b) ^31^P, c)^11^B, and d) ^1^H MAS NMR spectra of Li_3_PS_4_ + 2.5LiBH_4_ which were dried at various temperatures (drying temperatures are indicated in the spectra). All the spectra were measured at room temperature. Deconvolution results of the ^11^B MAS NMR spectra of Li_3_PS_4_ + 2.5LiBH_4_ dried at e) 50, f) 90, and g) 150 °C.

The impact of drying temperatures on the local structure of argyrodite, the presence of impurities, and the thermal decomposition have been investigated by solid‐state nuclear magnetic resonance (NMR) spectroscopy. As illustrated in **Figure** **4**a, the ^7^Li MAS NMR spectra of Li_5.25_PS_4.25_(BH_4_)_1.75_ (Li_3_PS_4_ + 2.5LiBH_4_) exhibit a single resonance at ≈0.2 ppm for the samples that are dried between 50 and 90 °C. As the drying temperature increases up to 90 °C, the linewidth gradually becomes narrower, suggesting that Li‐ion motion is enhanced and reaches maximum mobility in the sample dried at 90 °C. This trend is consistent with the ionic conductivity (Figure 2b), which shows an increase in ionic conductivity, reaching a maximum in the sample dried at 90 °C, followed by a subsequent decrease when dried above this temperature. As the drying temperature is further increased, the ^7^Li NMR signal gradually shifts to a higher frequency with significant line broadening. The sample dried at 370 °C, two broad signals at 2.3 and 1.0 ppm appear, indicating that a complete structural change has occurred at high temperature and that the breakdown of the structure results in the formation of by‐products such as Li_2_S.

Similar trends were observed in the ^31^P and ^11^B MAS NMR spectra (Figure 4b,c). One dominant ^31^P NMR signal at 90 ppm is present for the samples dried between 50 and 90 °C, whereas an appearance of an additional signal and line broadening is observed for the samples dried at higher temperatures exceeding 90 °C. ^31^P NMR spectra can provide valuable information about the local structure surrounding phosphorous in the lithium thiophosphate system. For Li_2_S‐P_2_S_5_ glass, several building units have been reported such as [PS_4_]^3−^, [P_2_S_7_]^4−^, [P_2_S_6_]^4−^, yielding ^31^P MAS NMR signals at ≈87, ≈91, and ≈105 ppm, respectively.^[^ ^17^ ^]^ For Li_3_PS_4_, a signal at ≈87 ppm has been observed, which has been assigned to the [PS_4_]^3−^ building group.^[^ ^17^ ^]^ Moreover, when S^2−^ is partially substituted by secondary anion, ^31^P NMR frequency varies depending on the type of the anions and on the ordering between S^2−^ and secondary anions. For example, Li_6_PS_5_X (X = Cl, Br, I) exhibits ^31^P NMR signals at ≈84, ≈92, ≈96 ppm for X = Cl, Br, and I, respectively.^[^ ^4^, ^5^ ^]^ In our previous work of Cl^−^ and BH_4_^−^ substituted argyrodite, ^31^P NMR signal at ≈89 ppm has been seen, which was assigned to the [PS_4_]^3−^ unit of dual‐anion substituted argyrodite.^[^ ^9^ ^]^ Thus, the signal at ≈90 ppm can be easily attributed to the [PS_4_]^3−^ unit of BH_4_^−^ substituted argyrodite. Upon drying the samples at 130 °C, an additional strong signal emerges at ≈92 ppm. As the drying temperature is increased further, ^31^P NMR signal becomes broadened and shifts to a lower frequency, reaching ≈87 ppm at 330 °C drying. Since the formation of additional crystalline phases was not observed up to 330 °C drying (Figure S5i, Supporting Information), the appearance of an additional ^31^P NMR signal must be due to the change in the local structure or amorphous phase. It is likely that preferential loss of BH_4_^−^ at 4d site and variation in the BH_4_^−^ distribution at 4d/4a site depending on the drying temperature (see Figure S7, Supporting Information; Table 2) can cause changes in the local structure. There are several possible explanations for the additional signal at ≈92 ppm; 1) formation of [P_2_S_7_]^4−^‐like local structure,^[^ ^4^, ^17^ ^]^ 2) change in the distribution of anions in the second coordination shell of the [PS_4_]^3−^ unit. Previously, multiple discrete signals have been reported for Li_6‐x_PS_5‐x_Cl_1 + x_, which have been assigned to the [PS_4_]^3−^ unit surrounded by (S^2−^)_3_(Cl^−^)_1_, (S^2−^)_2_(Cl^−^)_2_, (S^2−^)_1_(Cl^−^)_3_, and (Cl^−^)_4_ in their second coordination shell.^[^ ^15a^ ^]^ The exact structural nature of multiple signals resulting from high‐temperature drying is not clear at present. However, apparently, drying the samples at elevated temperatures above 100 °C leads to decomposition and changes in the site distribution of BH_4_^−^, altering local structure of argyrodite and loss of BH_4_^−^ from the structure inducing gradual transformation into Li_7_PS_6_‐like structure. In ^11^B MAS NMR spectra, a dominant BH_4_^−^ signal is observed at −42 ppm, which remains at a similar position up to 330 °C. For the samples dried above 90 °C, the main signal starts to broaden and new peaks appear between −10 and −30 ppm. The emergence of new peaks is indicative of the thermal decomposition of BH_4_^−^‐containing argyrodite and the formation of byproducts, such as [B_10_H_10_]^2−^ and [B_12_H_12_]^2−^,^[^ ^18^ ^]^ as evidenced by the TGA‐MS data. At 370 °C, the materials decompose completely, resulting in the complete loss of P and BH_4_^−^, which is reflected in the disappearance of the corresponding NMR signals. Furthermore, the influence of drying temperature on the site distribution of BH_4_^−^ between 4a and 4d sites was examined in detail through the analysis of ^11^B MAS NMR spectra (Figure 4e–g). The deconvolution results of the ^11^B MAS NMR spectra indicate that the distribution of BH_4_^−^ at the 4a and 4d sites is similar until 90 °C drying. In this temperature range, BH_4_^−^ is found to occupy the 4a site with a slightly higher population than the 4d sites. At higher drying temperatures, the occupancy of the 4a site increases, while that of the 4d site decreases. This is consistent with the results that the distribution of BH_4_^−^ over the 4a and 4d sites varies depending on the drying temperature as shown by XRD analysis (see Figure S7, Table 2).

Furthermore, the ^1^H MAS NMR spectra (Figure 4d) were obtained. Keep in mind that the ball‐milled samples were dried by vacuum pumping at room temperature to remove the solvent before drying at high temperatures. The main broad signal of BH_4_^−^ at 0.3 ppm and the weak sharp peaks of o‐xylene at 2 and 6.8 ppm were observed. As previously discussed, the residual o‐xylene used as a solvent has the potential to reduce the ionic conductivity. Consequently, the heating and drying step was included to remove the residual solvent, with the aim of increasing the conductivity. Nevertheless, residual solvent peaks persist across all drying temperatures. This suggests that vacuum drying at room temperature can efficiently remove most of the solvent, but, a minor quantity of o‐xylene remains entrapped within the argyrodite particles, rendering its complete removal challenging. As the drying temperature is increased, the signal of BH_4_^−^ undergoes a reduction in intensity, with complete disappearance observed at 370 °C. Additionally, a new signal at 4.2 ppm appears above 150 °C, which is presumed to be by‐products generated through two‐step decomposition (Figure S5b, Supporting Information).

In conclusion, the structural transformation of the argyrodite sample (Li_3_PS_4_ + 2.5LiBH_4_) was demonstrated through solid‐state NMR spectroscopy upon drying at varying temperatures. The MAS NMR spectra exhibit narrow signals up to 90 °C, indicating the synthesis of an electrolyte with a well‐ordered crystal structure. Notably, no discernible alterations are observed in the site distribution of BH_4_^−^ and the residual o‐xylene content. Consequently, the observed maximum conductivity at 90 °C drying is postulated to result from other factors, such as changes in crystal size and interface structures. However, as the drying temperature exceeds 90 °C, the breakdown of the structure and formation of byproducts begin. It seems that the initiation of the thermal decomposition of BH_4_^−^ results in the transformation into a mixture of different local structures. From 290 °C, the decomposition is facilitated until the total loss of BH_4_^−^ and P at 370 °C.

### 2.4 Electrochemical Characterization of Li_5.25_PS_4.25_(BH_4_)_1.75_

**Figure 5:**
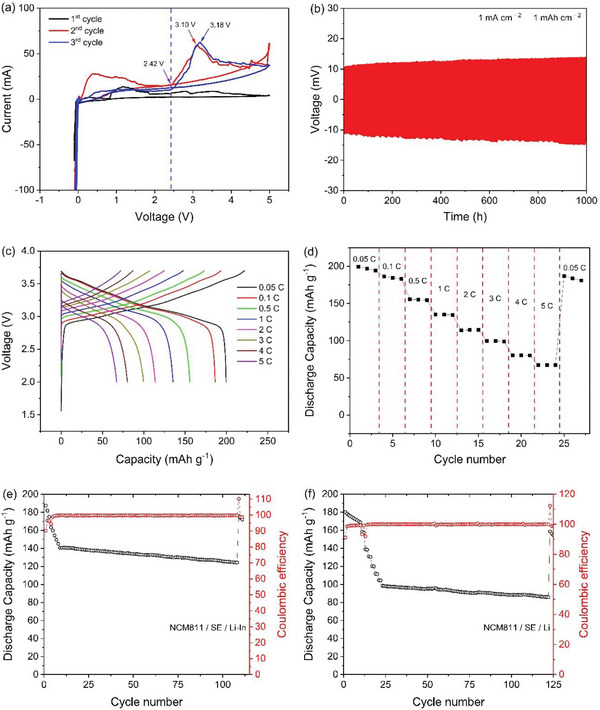
a) Cyclic Voltammetry (CV) data for the Li/solid electrolyte (Li_5.25_PS_4.25_(BH_4_)_1.75_)/C‐Li_5.25_PS_4.25_(BH_4_)_1.75_ cell. b) Voltage profiles of the Li metal symmetric cell (Li metal/solid electrolyte (Li_5.25_PS_4.25_(BH_4_)_1.75_)/Li metal). c) Voltage profile of the ASSB composed of Li_5.25_PS_4.25_(BH_4_)_1.75_ solid electrolyte (NCM811/solid electrolyte/Li‐In metal). d) Rate performance of Li_5.25_PS_4.25_(BH_4_)_1.75_ solid electrolyte (NCM811/solid electrolyte/Li‐In metal). e) The cycling performance results of the all‐solid‐state battery (ASSB) composed of Li_5.25_PS_4.25_(BH_4_)_1.75_ solid electrolyte (NCM811/solid electrolyte/Li‐In metal) for 100 cycles at 5C. The formation cycles consisted of charging and discharging at various rates: 0.05C, 0.1C, 0.2C, 0.5C, 1C (charge) and 0.5C (discharge), 2C (charge) and 0.5C (discharge), 3C (charge) and 0.5C (discharge), 4C (charge) and 0.5C (discharge) at constant current (CC). f) The cycling performance results of the all‐solid‐state battery (ASSB) composed of Li_5.25_PS_4.25_(BH_4_)_1.75_ solid electrolyte (NCM811/solid electrolyte/Li metal) for 100 cycles at 5C. The formation cycles consisted of charging and discharging at various rates: 0.1C (CCCV for 10 cycles), 0.2C, 0.5C, 1C (charge) and 0.5C (discharge), 2C (charge) and 0.5C (discharge), 3C (charge) and 0.5C (discharge), 4C (charge) and 0.5C (discharge) at constant current (CC).

After optimizing the synthesis conditions, we selected Li_5.25_PS_4.25_(BH_4_)_1.75_ dried at 90 °C with 7 mL of solvent using 10 mm balls, which has the highest ionic conductivity. To assess the electrochemical stability of the electrolyte, cyclic voltammetry (CV) was performed on Li_5.25_PS_4.25_(BH_4_)_1.75_. The measurements were taken over three cycles at a scan rate of 1 mV s^−1^ using a carbon‐electrolyte composite and Li metal. The electrolyte, Super P, and VGCF were mixed in a 70:15:15 ratio before use. The sweep speed of the CV test was 0.1 mV s^−1^. As shown in **Figure** **5**a, the main oxidation peak of the synthesized electrolyte was observed at 3.1 ≈3.2 V, with the onset of oxidation at ≈2.4 V. Both the onset and main peak occur at a slightly lower voltage compared to Li_6_PS_5_Cl.^[^ ^19^ ^]^

A Li symmetric cell was constructed and tested for 500 cycles under high constant current densities of 1 mA cm^−2^ to assess the Li deposition and dissolution behavior. Each deposition and dissolution step lasted for 1 h, totaling 1,000 h. Figure 5b illustrates the voltage profile of the symmetric cell. The overpotential exhibited negligible change throughout 500 cycles, and no additional side reactions were observed. This underscores the outstanding compatibility of the synthesized electrolyte with Li metal, demonstrating the absence of discernible side reactions or cell failures related to Li dendrite formation.

The capability of synthesized electrolytes to suppress dendrite formation was evaluated by measuring the critical current density (CCD). CCD represents the maximum current density that a symmetric cell can sustain through cycling without experiencing cell failure by short‐circuiting due to dendrite growth. Figure S9 (Supporting Information) displays the CCD profile of the cell with Li_5.25_PS_4.25_(BH_4_)_1.75_ as an electrolyte. An abrupt voltage drop observed at a current density of 2.5 mA cm^−2^ indicates a short circuit resulting from dendrite growth within the solid electrolyte, affirming the high CCD value. It is well‐established that materials with enhanced ionic conductivity tend to achieve a uniform current density distribution, thereby enhancing dendrite suppression capabilities.^[^ ^20^ ^]^ Therefore, it is believed that the high CCD value observed at 2.5 mA cm^−2^ correlates with the high ionic conductivity of the electrolyte.

To evaluate the electrochemical performance of the synthesized electrolyte, an all‐solid‐state battery was assembled with Li_5.25_PS_4.25_(BH_4_)_1.75_ electrolyte, lithium‐indium metal anode, and LiNbO_3_‐coated LiNi_0.8_Co_0.1_Mn_0.1_O_2_ cathode. The cell underwent cycling at different C‐rates within the voltage range between 2.0 and 3.7 V (versus Li^+^/Li–In) or between 2.7 and 4.3 V (versus Li^+^/Li) at 30 °C. Capacity calculations were based on the cathode's mass loading. Figure 5c illustrates the voltage profiles across different C‐rates. At 0.05C, the initial charge and discharge capacities were 208.0 and 187.6 mAh g^−1^, respectively, with an initial coulombic efficiency of 90.2% (Li‐In anode). The irreversible capacity is attributed to the formation of an interfacial layer between the argyrodite solid electrolyte and electrodes, a phenomenon well‐known in various studies on ASSBs.^[^ ^21^ ^]^ Figure 5d shows the rate performance of the cell. The cell performed rather well even at 5C (67.3 mAh g^−1^), indicating that the solid electrolyte works well even at higher rates. After 25 cycles, with increasing C‐rates up to 5C, the discharge capacity at 0.05C was 187.0 mAh g^−1^, indicating a capacity retention of 93.7%. This result indicates that the electrolyte performs well without significant decomposition even at higher C‐rates. The cycle performance at 5C is shown in Figure 5e. During the 100‐cycle tests, considering the demand for fast charging and relatively slower discharge rate in practical battery applications, the charge rate was varied from 1 to 5C, while the discharge rate was maintained at 0.5C. The charge and discharge capacity were well maintained up to 100 cycles without serious degradation. After 100 cycles, the discharge capacity at 0.05C was 176.2 mAh g^−1^, indicating a reversible discharge capacity retention of 93.9%. Furthermore, a full cell was tested using Li metal as the anode. Figure 5f shows the cycle performance of a cell composed of NCM811 and Li metal at 5C. The electrolyte exhibited minimal degradation even at the high charge rate of 5C over 100 cycles, with an initial coulombic efficiency of 91.08% during the 0.1C formation cycle. After 100 cycles at 5C, the discharge capacity at 0.1C was 158.70 mAh g^−1^, indicating a reversible discharge capacity retention of 94.0% compared to the discharge capacity at the 10th cycle.

## 3 Conclusion

In this study, we synthesized solid‐state electrolytes of BH_4_^−^ substituted argyrodite phase using a wet ball‐milling. The synthesized electrolytes exhibited a maximum conductivity of 13.8 mS cm^−1^ at room temperature when optimized wet ball‐milling parameters and vacuum drying temperature were employed. XRD analysis confirmed that the synthesized electrolytes had an argyrodite‐type structure, with non‐bridging S^2−^ replaced by BH_4_^−^ at both 4a and 4d sites in the structure. According to the results of Rietveld refinement, two non‐bridging S^2−^ ions in the argyrodite structure were replaced up to 1.75 out of a maximum 2. An ASSB using Li_5.25_PS_4.25_(BH_4_)_1.75_ as a solid electrolyte and LiNbO_3_‐coated NCM811 as cathode exhibited an initial discharge capacity of 187.6 mAh g^−1^, an initial Coulombic efficiency of 90.2% at 0.05C. The cell maintained 93.9% of its initial discharge capacity after 100 cycles at 5C. Li symmetric cells with Li_5.25_PS_4.25_(BH_4_)_1.75_ demonstrated stable Li plating and stripping cycling for over 1,000 h at 1 mA cm^−2^, along with a high critical current density of 2.5 mA cm^−2^. This wet mechanochemical process provides an efficient way to produce BH_4_‐substituted argyrodite‐type solid electrolytes, making it a lot more suitable for large‐scale applications compared to the high‐energy ball‐milling process followed by high‐temperature annealing.

## 4 Experimental Section

### Materials Preparation

Li_6−a_PS_5−a_(BH_4_)_1+a_ solid electrolytes were prepared using a wet ball‐milling method. Lithium sulfide (Li_2_S, Alfa–Aesar, 200 mesh, 99.9%), phosphorus pentasulfide (P_2_S_5_, Sigma–Aldrich, 99%), and lithium borohydride (LiBH_4_, Acros Organics, 95%) powders were used as starting materials. First, Li_2_S and P_2_S_5_ were mixed in a 3:1 molar ratio and placed into a 120 cm^3^ zirconia bowl with 45 zirconia balls (10 mm in diameter) and milled at 650 rpm for 2 h using a planetary ball mill (Retsch PM200). 2.5 wt% toluene was added as a process control agent to prevent excessive sticking of powder around the bottom corner of the bowl during milling. Second, LiBH_4_ was charged into the bowl together with o‐xylene. To mitigate temperature increase within the milling bowl, the milling process was interrupted for 15 min following every 5 min of milling (PULVERISETTE 7 premium line). The total run time for wet milling is 8 h but the actual milling time is only 2 h. For dry ball‐milling, all milling parameters were the same, except for the absence of solvent. The samples were handled in an Ar‐filled glovebox (O_2_ and H_2_O levels < 0.1 ppm). The wet‐milled samples were charged into a small stainless reactor tube and dried under a dynamic vacuum at preset temperatures for 2 h. The vacuum was maintained below 5 × 10^−3^ torr.

### X‐ray Powder Diffraction (XRD) Analysis

XRD analysis (D8 ADVANCE, Bruker AXS, with Cu Kα radiation (λ = 1.540598 Å)) was performed over the 2θ range of 10°–90° with a step size of 0.028° to analyze the crystal structure. Because of the air sensitivity, all the samples were sealed using a 7.5‐µm‐thick polyimide film with a lab‐made sample holder in the glove box.

### Rietveld Refinement Analysis

Rietveld refinement was performed using TOPAS v.5 software (Bruker AXS) over the 2θ range of 10°–90°. Because the limited quality of the XRD data did not allow for refining the position of H in BH_4_^−^, the B–H bond length was fixed to 1.1 Å, the usual bond length of B–H obtained from XRD. Within the symmetry of F−43m, there were two possible orientations of BH_4_^−^, and an equal occupancy for the two orientations was assumed. The assumptions and conditions applied during Rietveld refinement were consistent with those used in previous studies.^[^ ^9^ ^]^

### Thermal Gravimetric Analysis‐Mass Spectrometry (TGA‐MS)

TGA analysis was conducted using a Netzsch TG 209 F1 to analyze the progress of decomposition as a function of temperature for the synthesized electrolyte. ≈7–10 mg of the samples were sealed in an aluminum crucible, and a hole was drilled immediately before measurement to minimize contact with the atmosphere. Argon flowed at a rate of 20 cm^3^ min^−1^, and the temperature was increased to 400 °C at a rate of 10 K min^−1^. At the same time, mass spectrometer analysis was utilized for qualitative analysis of the decomposition products (HPR‐20 R&D, HIDEN). During the measurement, the vacuum level of the mass spectrometer was maintained between 2.5–5.0 × 10^−6^ torr.

### Electrochemical Impedance Spectroscopy (EIS), Electronic Conductivity Measurement (Chronoamperometry Measurement)

With an SS (SUS440C)/solid electrolyte/SS cell, the ionic conductivity was measured using EIS (IviumStat.h, Ivium Technologies). A 120 mg sample was placed into a 6‐mm diameter mold made of zirconia, cold‐pressed at 566 MPa, and then, measurements were performed at 188 MPa and 25 °C. The frequency range was from 1 MHz to 100 Hz at an amplitude of 100 mV. For the electronic conductivity measurements, 90 mg of the electrolyte was placed in a 13 mm diameter mold and cold‐pressed at 100 MPa. Afterward, 20 µm thick SUS 430 foil was placed on both the top and bottom of the sample, and measurements were conducted at 25 °C under 20 MPa using a Solartron 1287 (Solartron Instruments, UK). The measurements were conducted by maintaining a constant voltage of 0.1 mV.

### Solid‐State NMR

Magic Angle Spinning (MAS) NMR experiments were conducted using Bruker AVANCE NEO 600 MHz spectrometer with a spinning frequency of 22 kHz. ^7^Li MAS NMR experiments were performed at a resonance frequency of 232.2 MHz, 90° pulse of 3.4 s, and a relaxation delay of 10 s. ^31^P NMR spectra were obtained at a resonance frequency of 242.9 MHz, 90° pulse length of 3.75 s, and a relaxation delay of 60 s. ^11^B NMR spectra were recorded at 192.5 MHz applying TRIP pulse sequence to remove the background signal with 90° pulse length of 1 s and a relaxation delay of 2 s. ^1^H NMR spectra were recorded with an operating frequency of 599.97 MHz, 90° pulse length of 2.5 s, and relaxation delay of 5 s. LiCl (1 m aq.), H_3_PO_4_, boric acid, and tetramethylsilane (TMS) were used as the chemical shift reference at 0.0, 0.0, 19.4, and 0 ppm for ^7^Li, ^31^P, ^11^B, and ^1^H NMR, respectively.

### Cyclic Voltammetry (CV) Measurement

CV was conducted to evaluate the electrochemical stability of the solid electrolyte (SE). First, 110 mg of the electrolyte was pressed at 396 MPa into a polyether ketone (PEEK) mold to form a pellet. The working electrode was 10 mg of C–Li_5.25_PS_4.25_(BH_4_)_1.75_ (with electrolyte, Super P, and VGCF were mixed in a 70:15:15 ratio), and the counter/reference electrode was 9‐mm diameter Li metal sheet affixed to the top side (Li/SE/C‐SE). The CV curves were obtained using a potentiostat (VMP3, BioLogic) between −0.1 and 5.0 V at 30 °C and a scan rate of 1 mV s^−1^, repeated 3 times.

### Lithium Symmetric Cell and Critical Current Density (CCD) Measurement

A Li symmetric cell was assembled to test the lithium deposition and dissolution, and 500 cycles were performed for 1 h each (WBCS3000L, WonATech). The as‐synthesized electrolyte powder (110 mg) was placed into a 10 mm‐diameter mold and cold‐pressed at 396 MPa. Then 9‐mm‐diameter Li‐metal sheets were placed on both sides of the mold (Li/SE/Li). Finally, the cell was assembled under a constant torque of 7 N m and electrochemical properties were measured at 30 °C. The applied current density was 1 mA cm^−2^. The critical current density was measured after assembling in the same method as the Li symmetric cell. The time for each charge and discharge was 1 h, and the step size for the current increase was 0.1 mA cm^−2^.

### Fabrication and Characterization of ASSB Cells

Metal sheets consisting of Li foil served as the anode for the cell test. The cathode composites were composed of LiNbO_3_‐coated LiNi_0.8_Co_0.1_Mn_0.1_O_2_ (80 wt%), a conducting agent (Super P, 1 wt%), and the solid electrolyte (19 wt%). For the cell assembly, the synthesized solid‐electrolyte powders (110 mg) were placed in a PEEK mold with a 10‐mm diameter and then pressed at 368 MPa. The cathode composites (9 mg) were added on top of the solid‐electrolyte pellet with aluminum foil (10‐mm diameter, current collector) and pressed at 425 MPa. The other side was an indium foil (9‐mm diameter, 2‐mm thickness) and lithium foil (4‐mm diameter, 2‐mm thickness) anode with copper foil (10‐mm diameter, current collector). Finally, the mold was assembled under a constant torque of 10 N m. The cells were assembled in the glovebox and galvanostatically tested at various current densities at 30 °C under normal atmospheric conditions with sealing. Based on the well‐known theoretical specific capacity of NCM811 (≈200 mAh g^−1^), the practical capacity was calculated to be 200 mAh g^−1^. And 1C was calculated based on the total mass of the cathode mixture (80 wt% of 9 mg): 1C = 200 mAh g^−1^ × 0.009 g × 80 wt% / 0.7854 cm^2^ = 1.83 mA cm^−2^. A battery cycler (WBCS3000L, WonATech) was used to determine the ASSB rate properties and for cycling testing and had a voltage cut‐off range between 2.0 and 3.7 V (as compared to Li^+^/Li–In). The electrochemical performance was characterized at 0.05, 0.1, 0.2, 0.5, 1.0, 2.0, 3.0, 4.0 and 5.0 C. As shown in the previous study,^[^ ^9^ ^]^ the electrode thickness was 1.6 mm at the anode and 55 µm at the cathode.

## Conflict of Interest

The authors declare no conflict of interest.

## Supporting information

Supporting Information

## Data Availability

The data that support the findings of this study are available from the corresponding author upon reasonable request.
