# Supplementary material for: Wet Mechanochemical Synthesis of BH4‐Substituted Lithium Argyrodites
Source: Small Methods. 2024 Sep 5;9(3):2401046. doi: 10.1002/smtd.202401046 (PMC11926510; doi:10.1002/smtd.202401046)
Supplement: Supplementary file 1 — Supporting Information [file SMTD-9-2401046-s001.docx]

**Supporting Information**

**Wet mechanochemical synthesis of BH_4_-substituted lithium argyrodites**

*Ji-Hoon Han, Yoonju Shin, Young Joo Lee, Sangdoo Ahn, Young-Su Lee, Kyung-Woo Yi, Young Whan Cho**

J.-H. Han, Y.-S. Lee, Y. W. Cho

Energy Materials Research Center, Korea Institute of Science and Technology (KIST), Seoul 02792, Republic of Korea

J.-H. Han, K.-W. Yi

Department of Materials Science and Engineering, Seoul National University, Seoul 08826, Republic of Korea

Yoonju Shin, Young Joo Lee

Metropolitan Seoul Center, Korea Basic Science Institute (KBSI), Seoul 03759, Republic of Korea

Yoonju Shin, Sangdoo Ahn, Young Joo Lee

Department of Chemistry, Chung-Ang University, Seoul 06974, Republic of Korea

* Corresponding author

E-mail: [oze@kist.re.kr](mailto:oze@kist.re.kr)

**
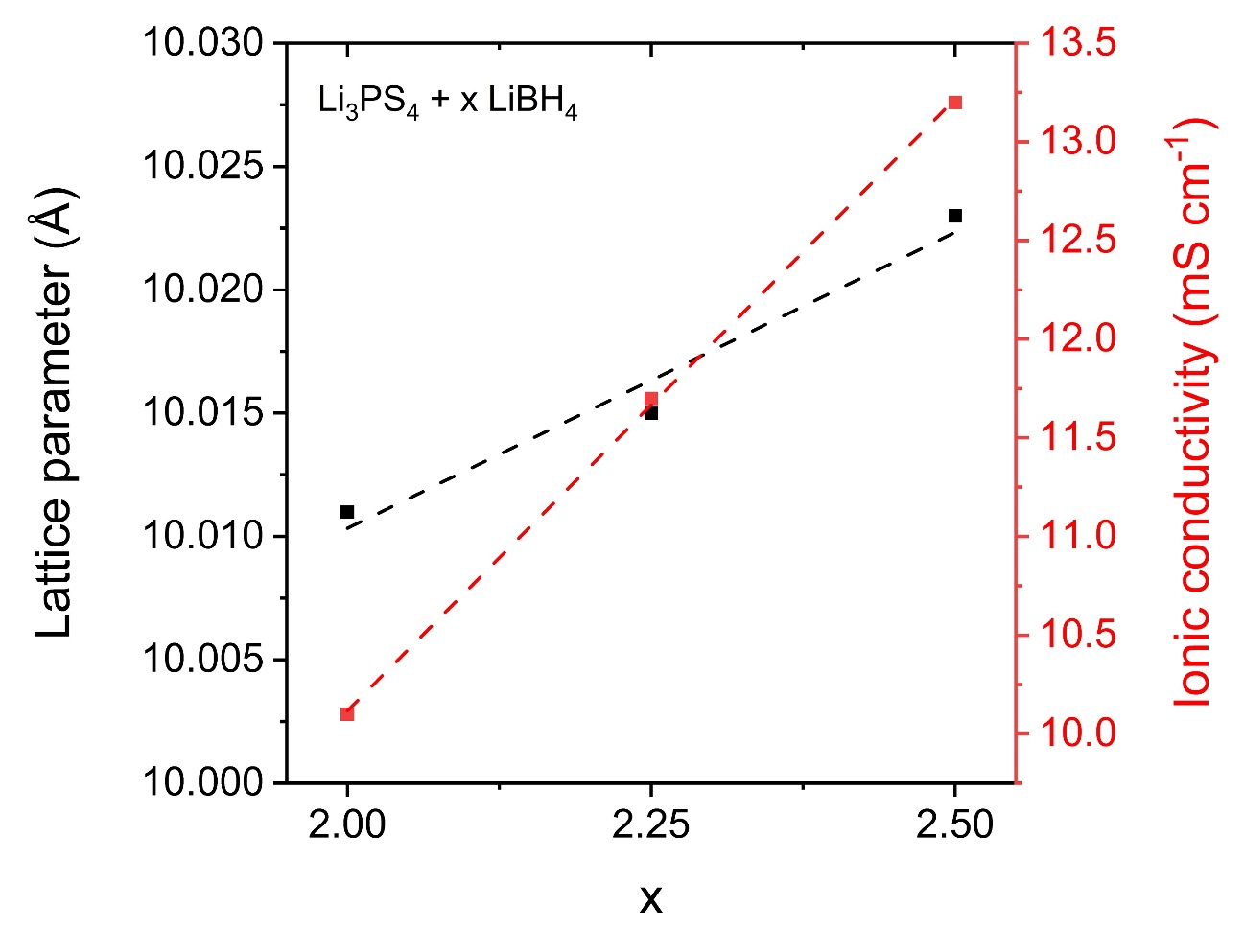
**

**Figure S1.** The trend of ionic conductivity and lattice parameter changes as a function of x in Li_3_PS_4­_ + xLiBH_4_. As more LiBH_4_ is added, both the ionic conductivity and lattice parameter increase. This is attributed to the increased substitution of BH_4_^−^ in the argyrodite structure.

**
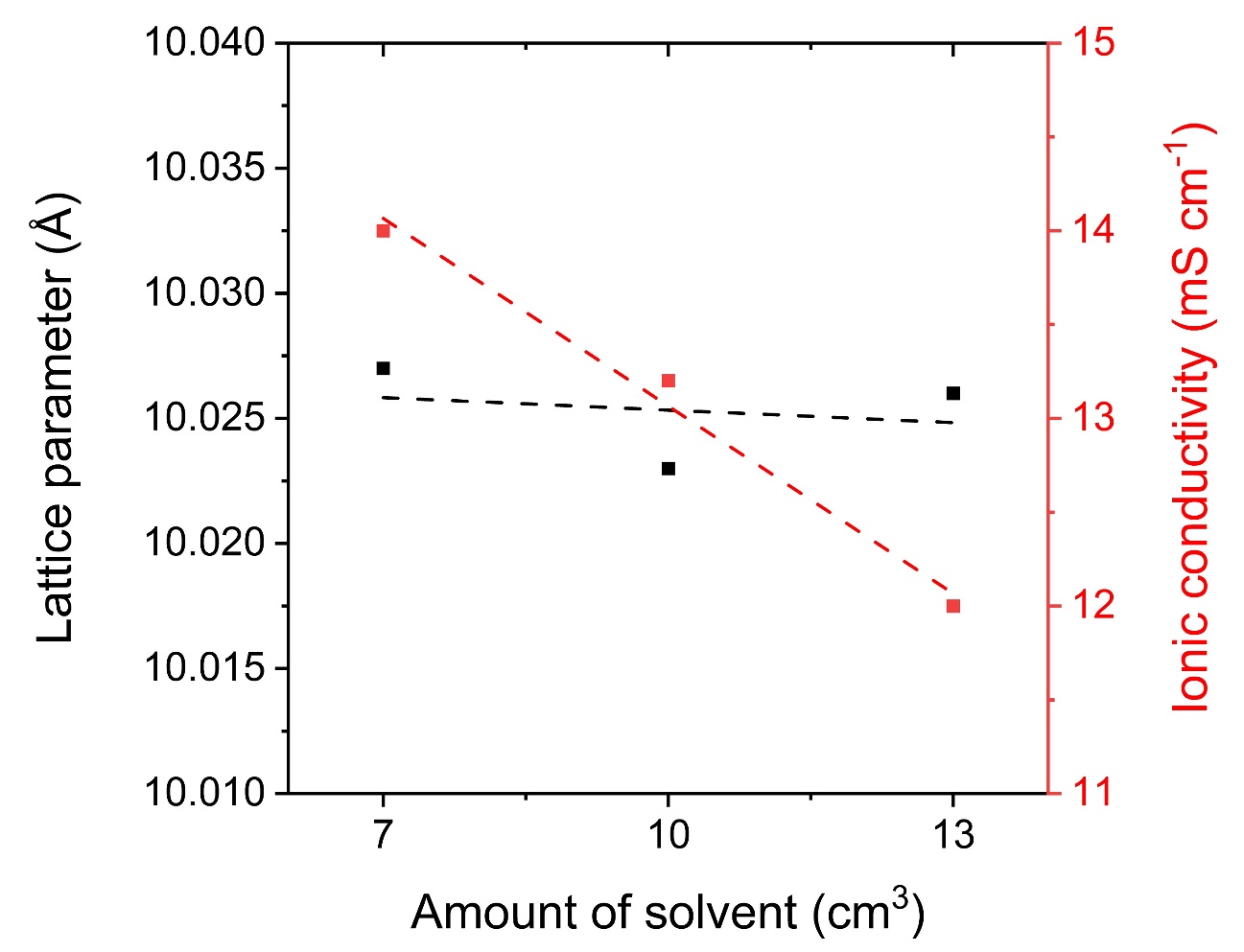
**

**Figure S2.** The trend of ionic conductivity and lattice parameter changes with the amount of solvent used in wet milling. While the chemical composition remains constant, there is minimal change in lattice parameter. However, ionic conductivity showed an inverse relationship with the amount of solvent used.

**
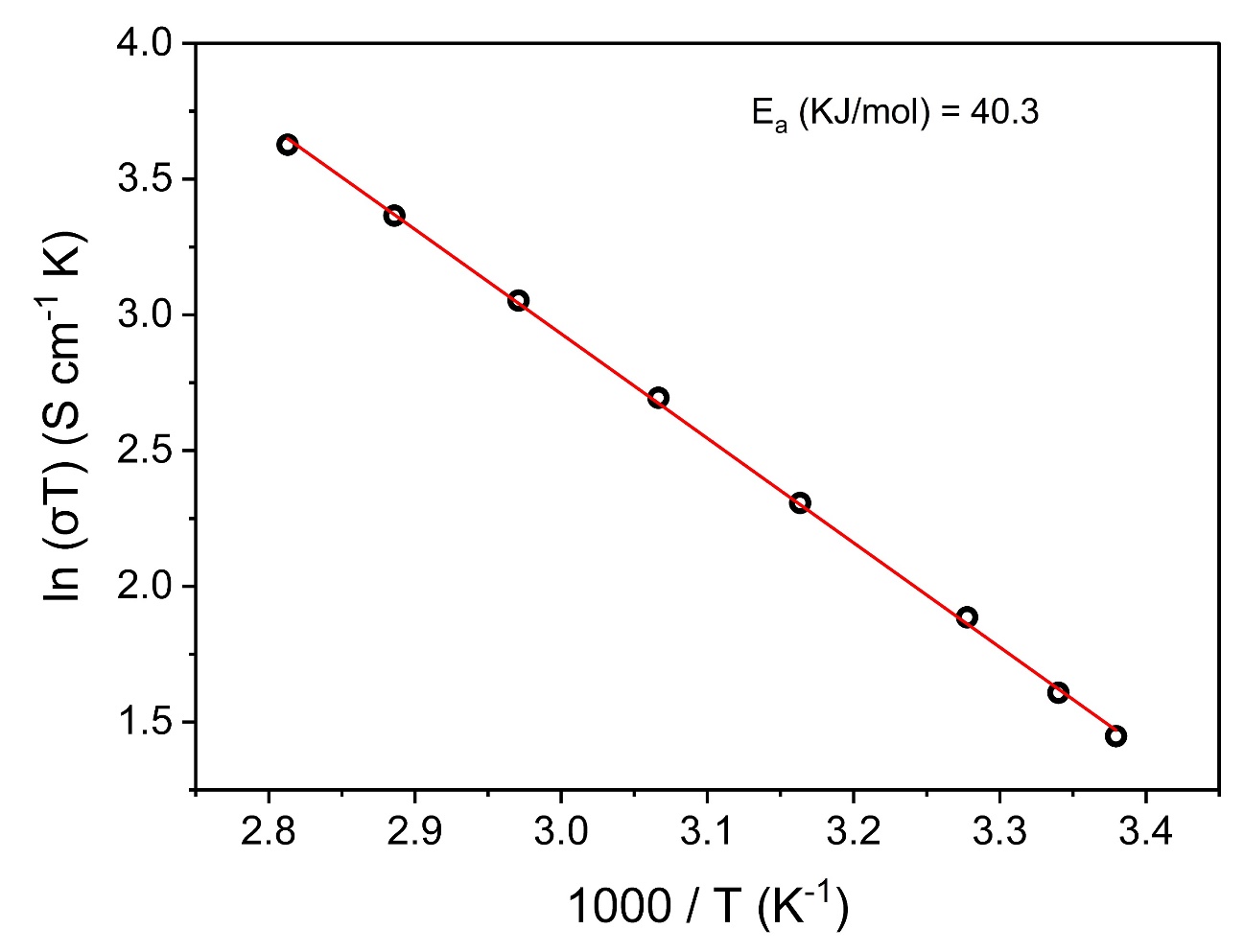
**

**Figure S3.** The activation energy of Li_5.25_PS_4.25_(BH_4_)_1.75_ after vacuum drying at 90 °C. It was determined by measuring the ionic conductivity as a function of temperature and calculating the activation energy using the Arrhenius equation. The activation energy was measured to be 40.3 kJ mol^−1^.


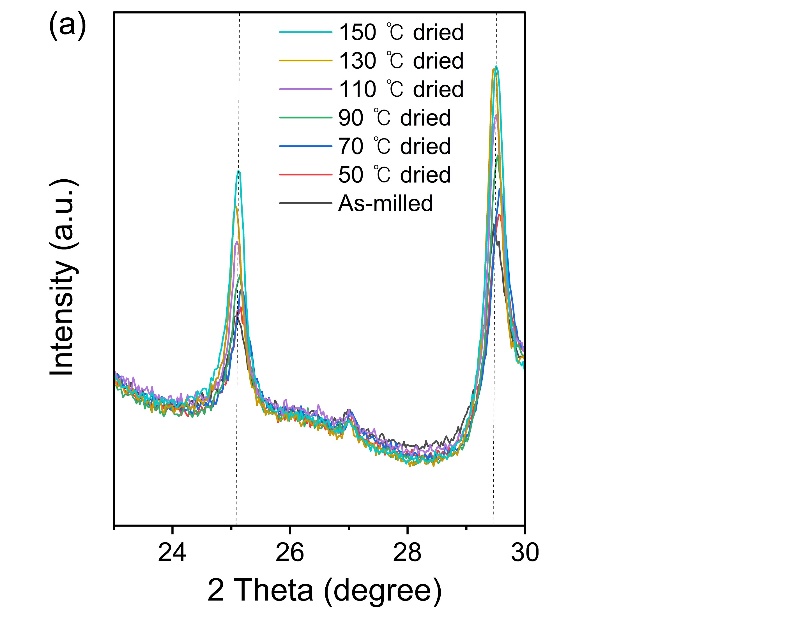

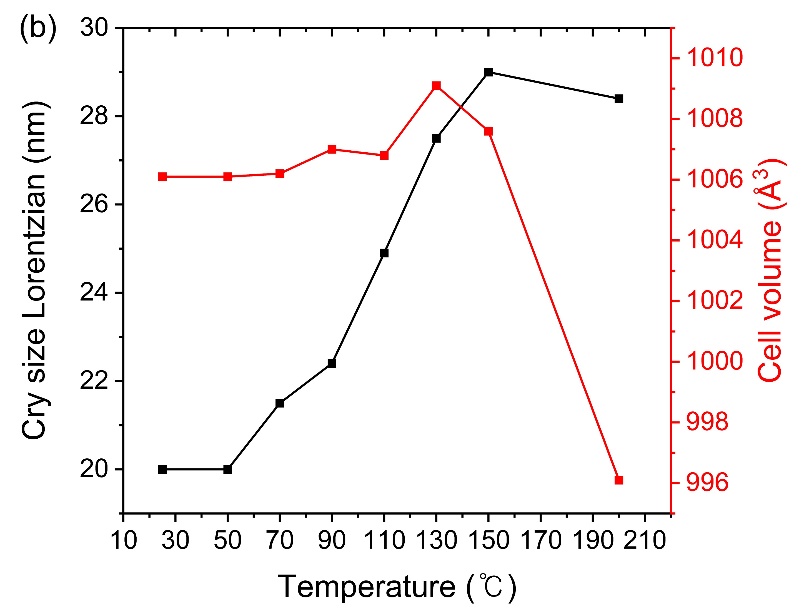


**Figure S4.** (a) The intensity variation of XRD peaks of Li_5.25_PS_4.25_(BH_4_)_1.75_ as a function of drying temperature. While the intensity variation of the Li_2_S peak near 27 degrees was not significant, the intensity of the argyrodite peak increased proportionally with increasing drying temperature. (b) The cell volume and crystal size versus drying temperature.

**
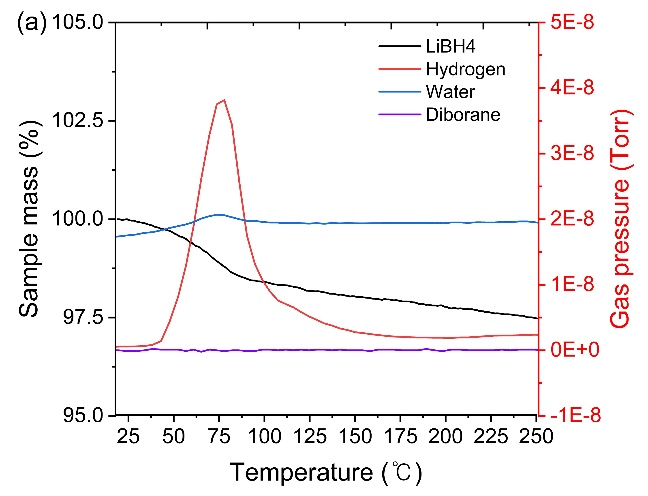

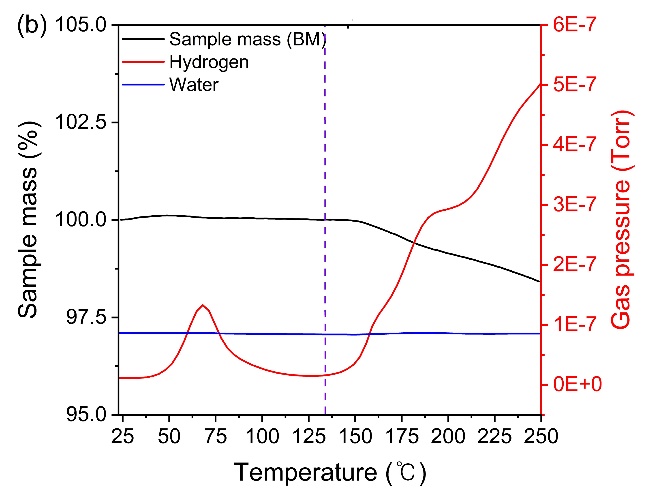

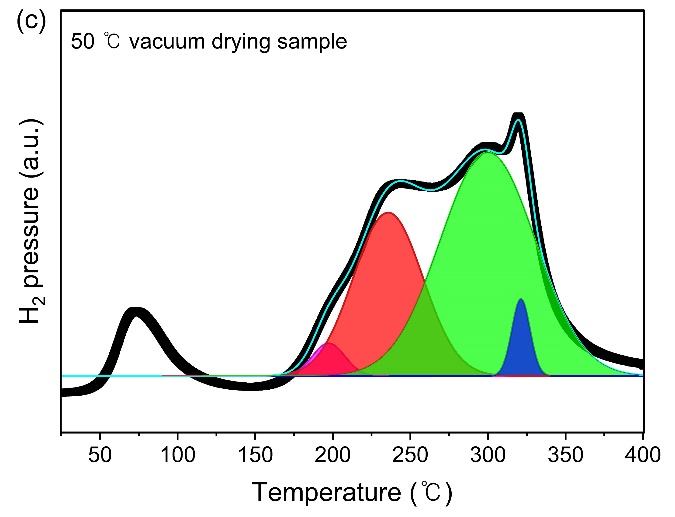

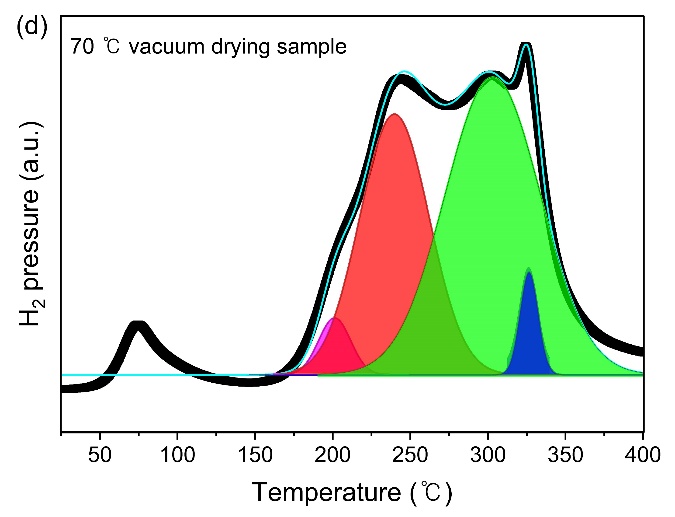

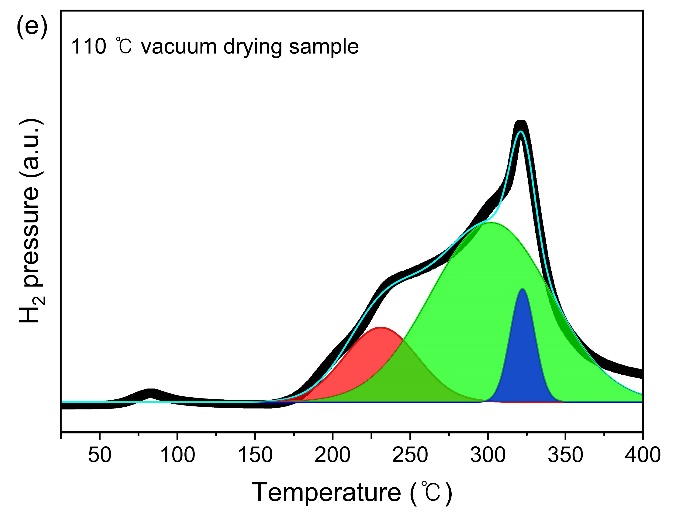

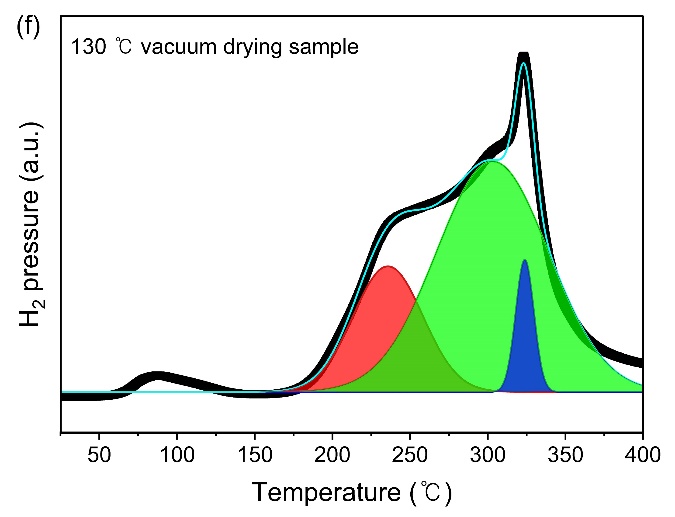
**

**
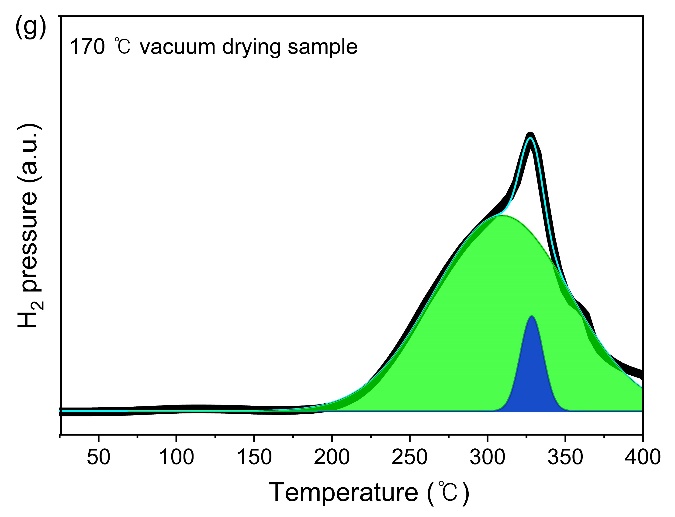

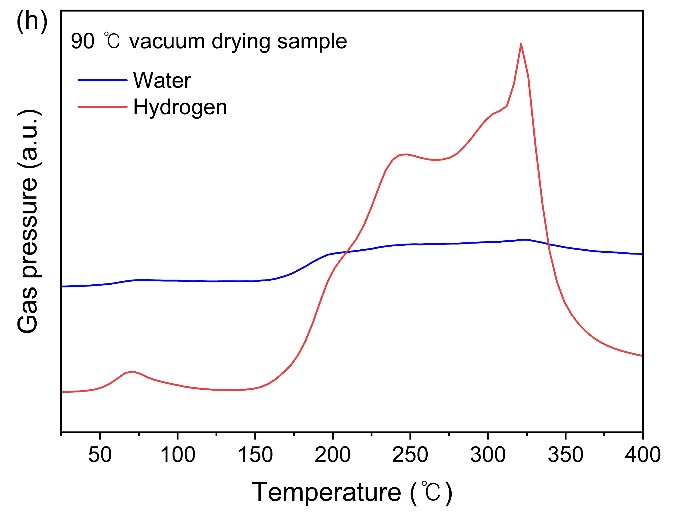
**

**
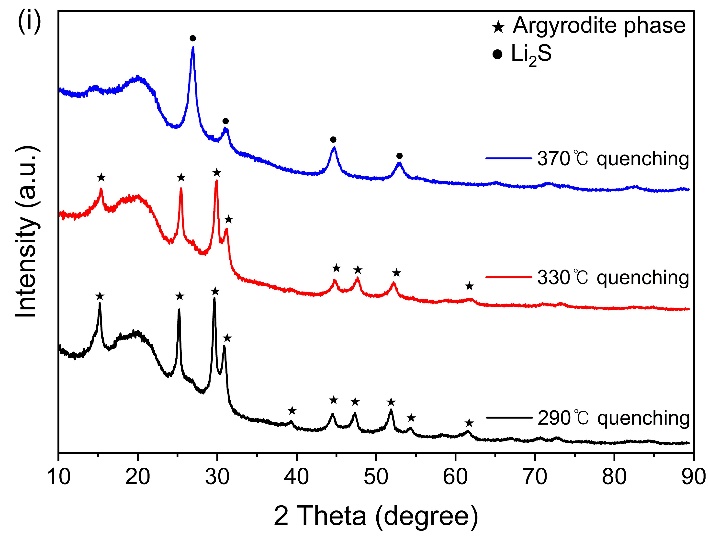
**

**Figure S5.** (a, b) Thermogravimetric analysis coupled with mass spectrometry (TGA-MS) data under heating rate of 5 K min^−1^ for (a) the pristine LiBH_4_ and (b) the solid electrolyte synthesized via dry ball-milling, respectively. (c-g) H_2_ profiles (MS data) under heating rate of 10 K min^−1^ for the samples dried at (c) 50, (d) 70, (e) 110, (f) 130, and (g) 170 °C. (h) H_2_ and water profiles (MS data) under heating rate of 10 K min^−1^ for the sample dried at 90 °C. (i) The XRD data measured at room temperature after heating the samples to 290 °C, 330 °C, and 370 °C at a heating rate of 10 K min^−1^ followed by quenching in liquid nitrogen.

From the hydrogen peaks in the LiBH_4_ data, it can be inferred that the peak around 70 °C, present in all TGA-MS datasets, originates from impurities contained in the pristine LiBH_4_. Above 150 °C, almost same hydrogen peaks appear regardless of the use of solvent (b-h), indicating decomposition of BH_4_-substituted argyrodite under Ar flowing condition. In panels c to g, the H_2_ profile was deconvoluted into several peaks. The red and green peak correspond to the decomposition of BH_4_^−^ at the 4d and 4a site, respectively. Observing **Figure S5h**, the pink peak shown in **S5c-d** seems to be associated with H_2_O. Looking at **Figure S5i**, the sample quenched at 330 °C maintained the argyrodite phase, whereas the sample quenched at 370 °C completely decomposed into Li_2_S and other amorphous phase(s). Therefore, the blue peak shown in **S5c-g** is associated with the decomposition of the argyrodite phase, and it is presumed that the remaining BH_4_^−^ decomposes at once as the structure collapses.

**
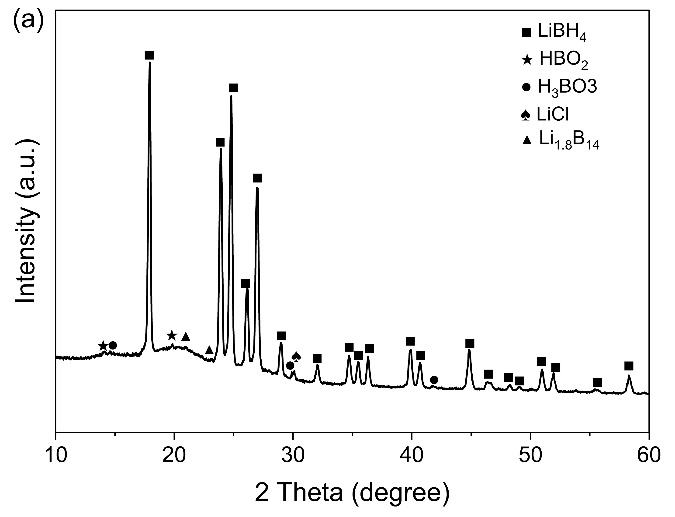

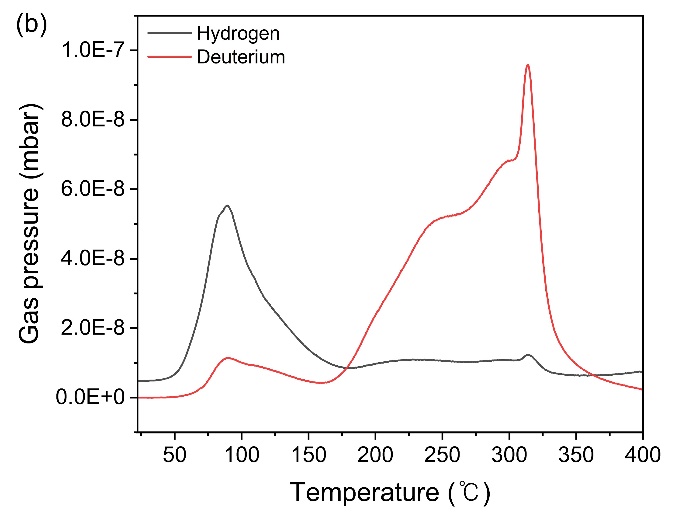
**

**Figure S6.** (a) XRD data of the LiBH_4_ reagent. Peaks corresponding to HBO_2_, H_3_BO_3_, and LiCl, in addition to LiBH_4_, were detected. (b) Mass spectrometry data of the Li_3_PS_4_+2.5LiBD_4_ sample. A significant hydrogen peak was detected below 100 °C, but the deuterium peak was minimal, indicating that this peak did not originate from the argyrodite. Instead, it originated from substances like HBO_2_ and H_3_BO_3_, which formed due to the electrolyte reacting with ambient moisture.


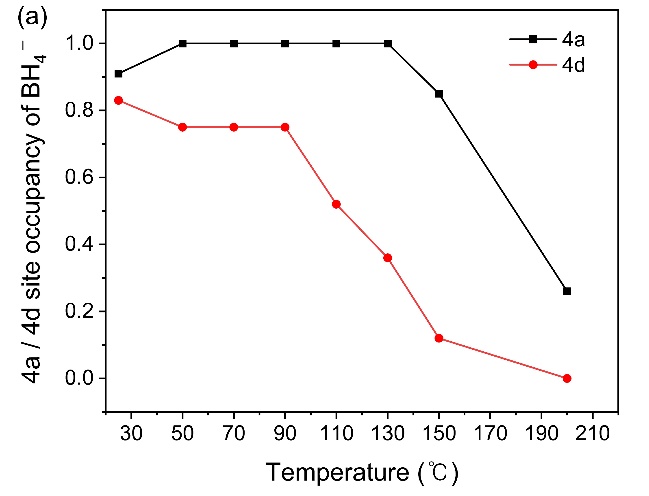

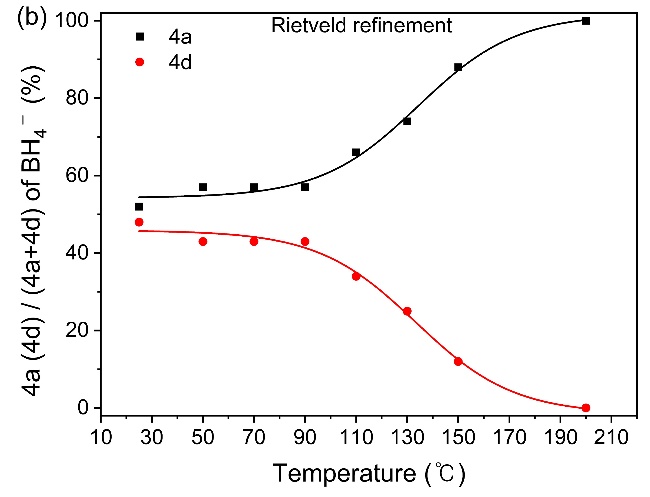


**
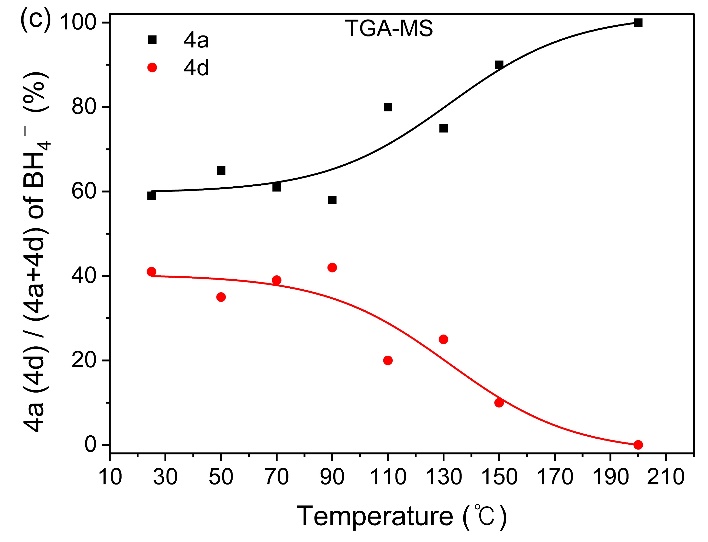
**

**Figure S7.** (a) Site occupancy of BH_4_^−^ at the 4a and 4d sites of argyrodite. Site occupancy of 4d site starts to decrease with temperature at much lower temperature than that of 4a site. (b) Relative ratio of BH_4_^−^ at the 4a (or 4d) site to the total substitution amount (4a+4d) calculated by Rietveld refinement. (c) Relative ratio of BH_4_^−^ at the 4a (or 4d) site to the total substitution amount (4a+4d) estimated by the area of deconvoluted peaks in the MS profile of H_2_.

**
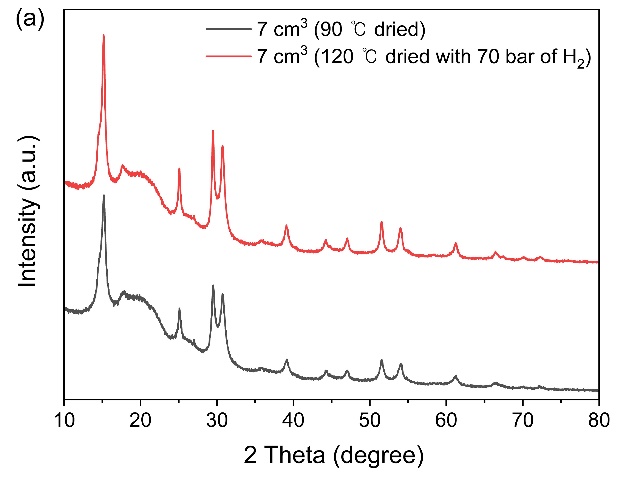

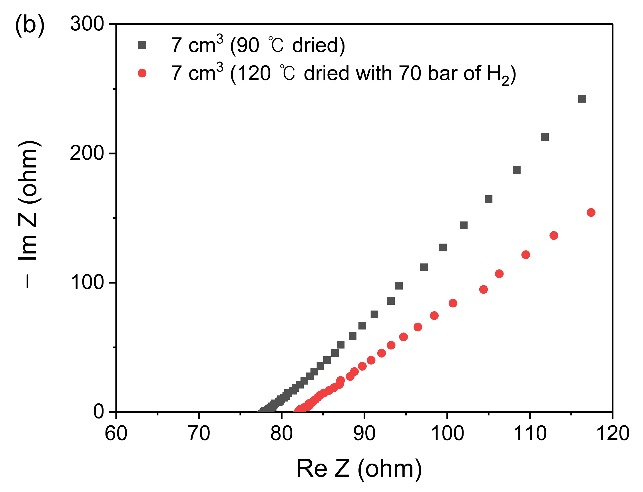
**

**Figure S8.** (a) XRD profile and (b) ionic conductivity of the electrolytes dried at 90 °C under vacuum and at 120 °C under 70 bar of hydrogen pressure. The resistance of the electrolyte heated at 120 °C under 70 bar of hydrogen pressure is higher than that of the electrolyte dried at 90°C under vacuum, indicating that BH_4_-substituted argyrodite decomposes at temperatures above 120 °C even under high hydrogen pressure.


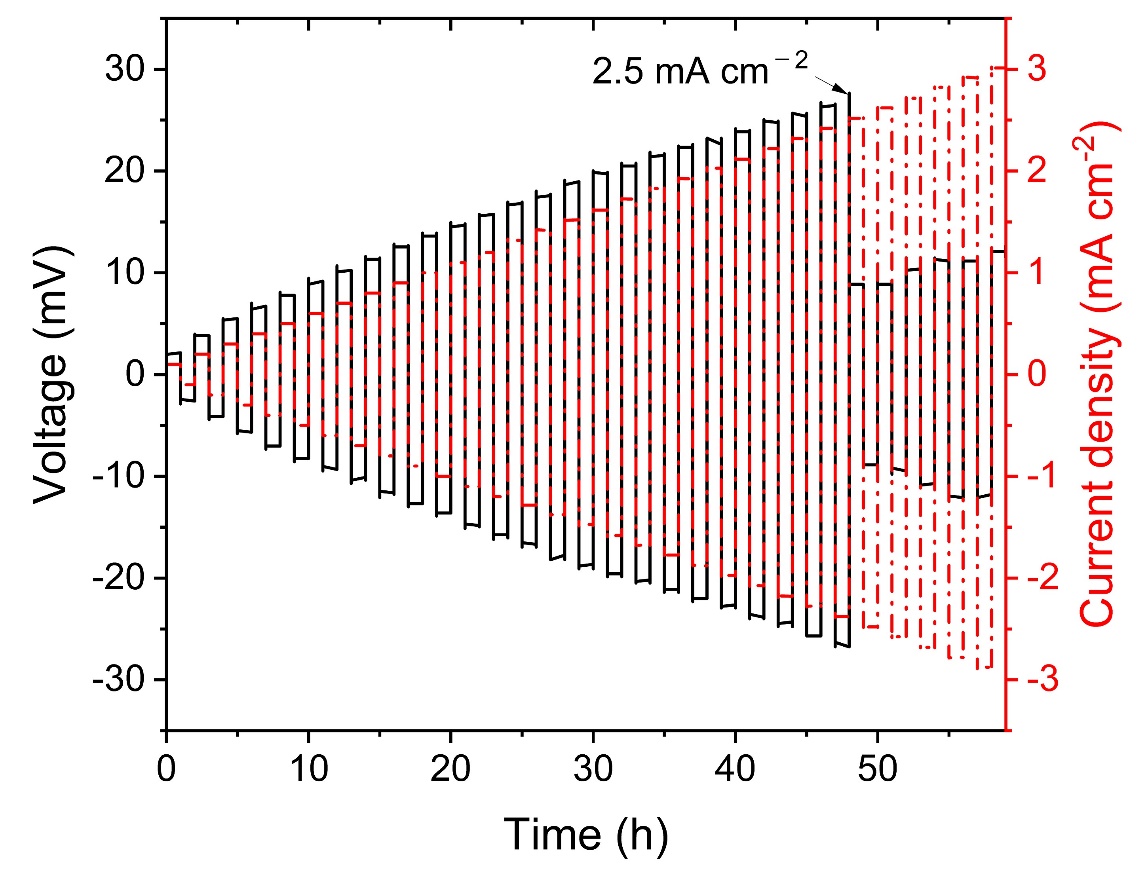


**Figure S9**. The Critical Current Density (CCD) data for Li_5.25_PS_4.25_(BH_4_)_1.75_. The voltage decreased at 2.5 mA cm^−2^, indicating that the CCD for this electrolyte is 2.5 mA cm^−2^. The critical current density was measured after assembling in the same method as the Li symmetric cell. The time for each charge and discharge is 1 h, and step size for the current increase is 0.1 mA cm^−2^.


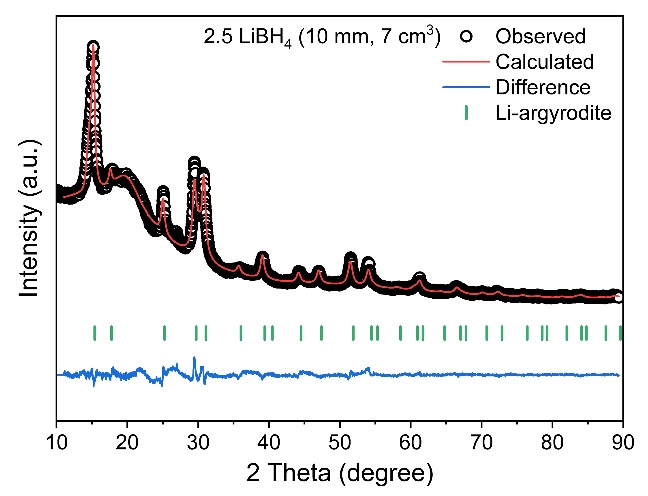

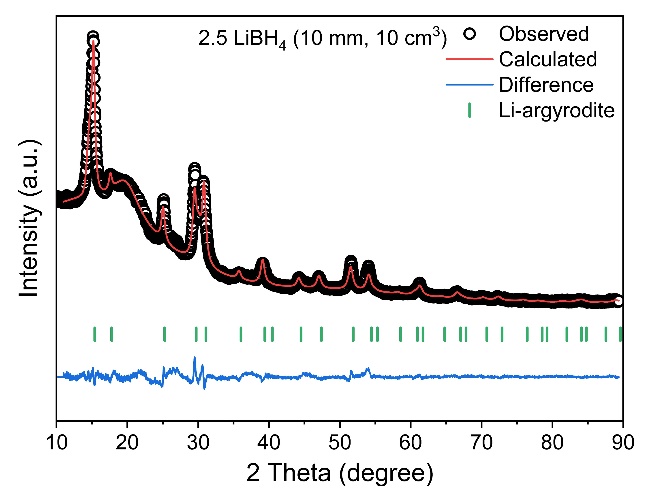

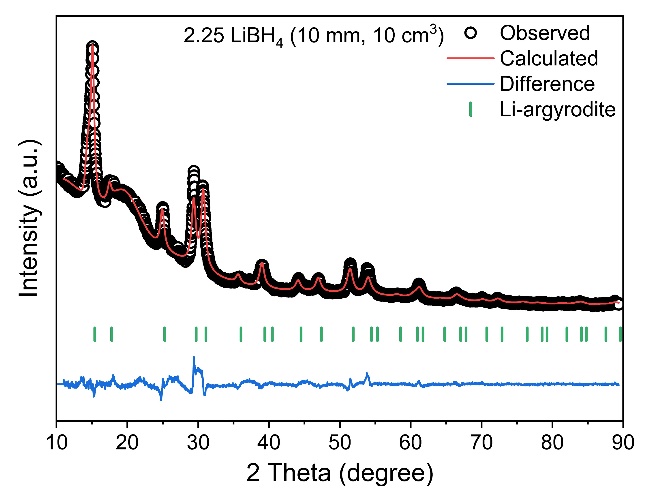

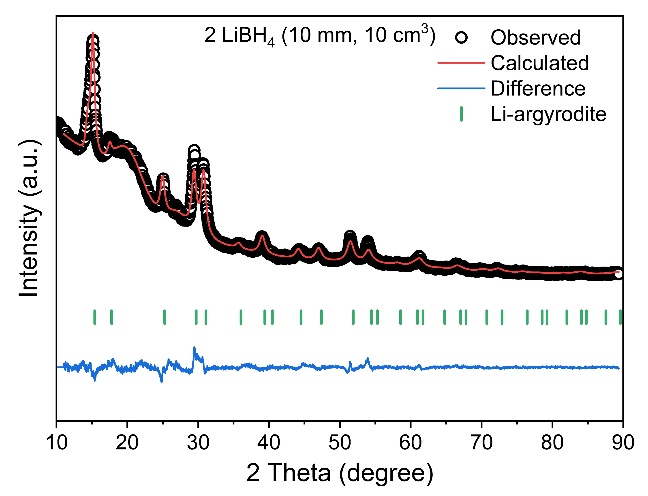

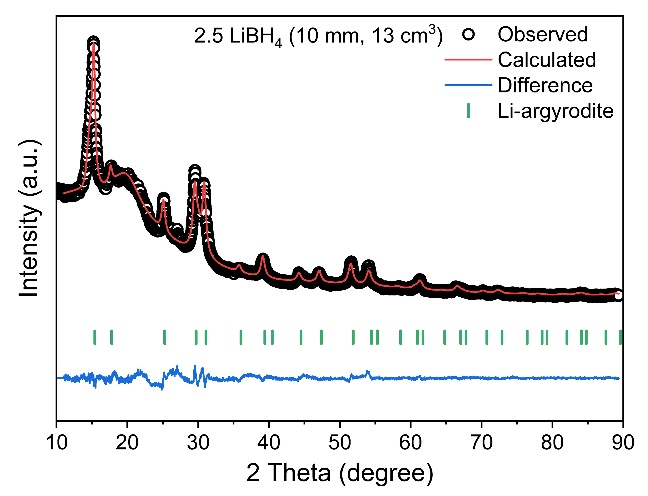


**Figure S10**. Rietveld refinement results of the electrolyte synthesized under various conditions. In each panel, the synthesis condition is annotated. For example, “2.5 LiBH_4_ (10 mm, 7 cm^3^)” represents that the powders were prepared in the mixing ratio of Li_3_PS_4_+2.5LiBH_4_ and wet ball-milled using 10-mm balls and 7 cm^3^ of o-xylene.


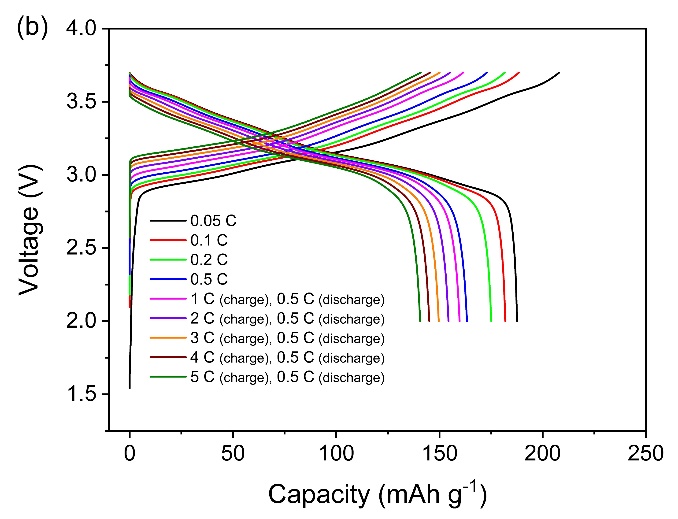

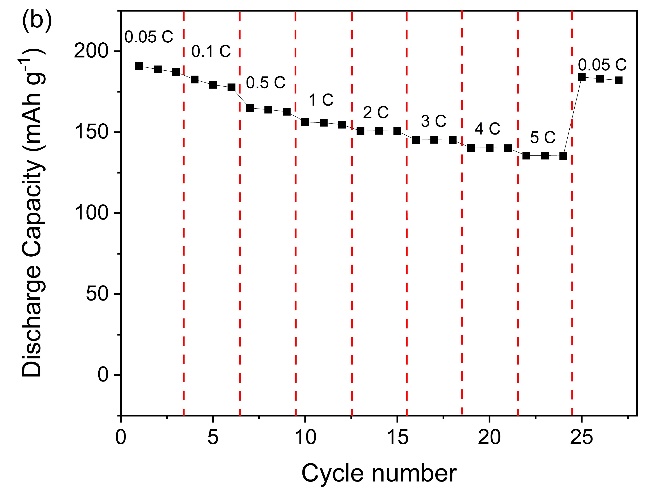


**Figure S11**. (a) The voltage profile and (b) rate performance of the all-solid-state battery (ASSB) were evaluated when charged at 1C, 2C, 3C, 4C, and 5C rates, and discharged at a fixed rate of 0.5C (when the charging rate is above 1C).


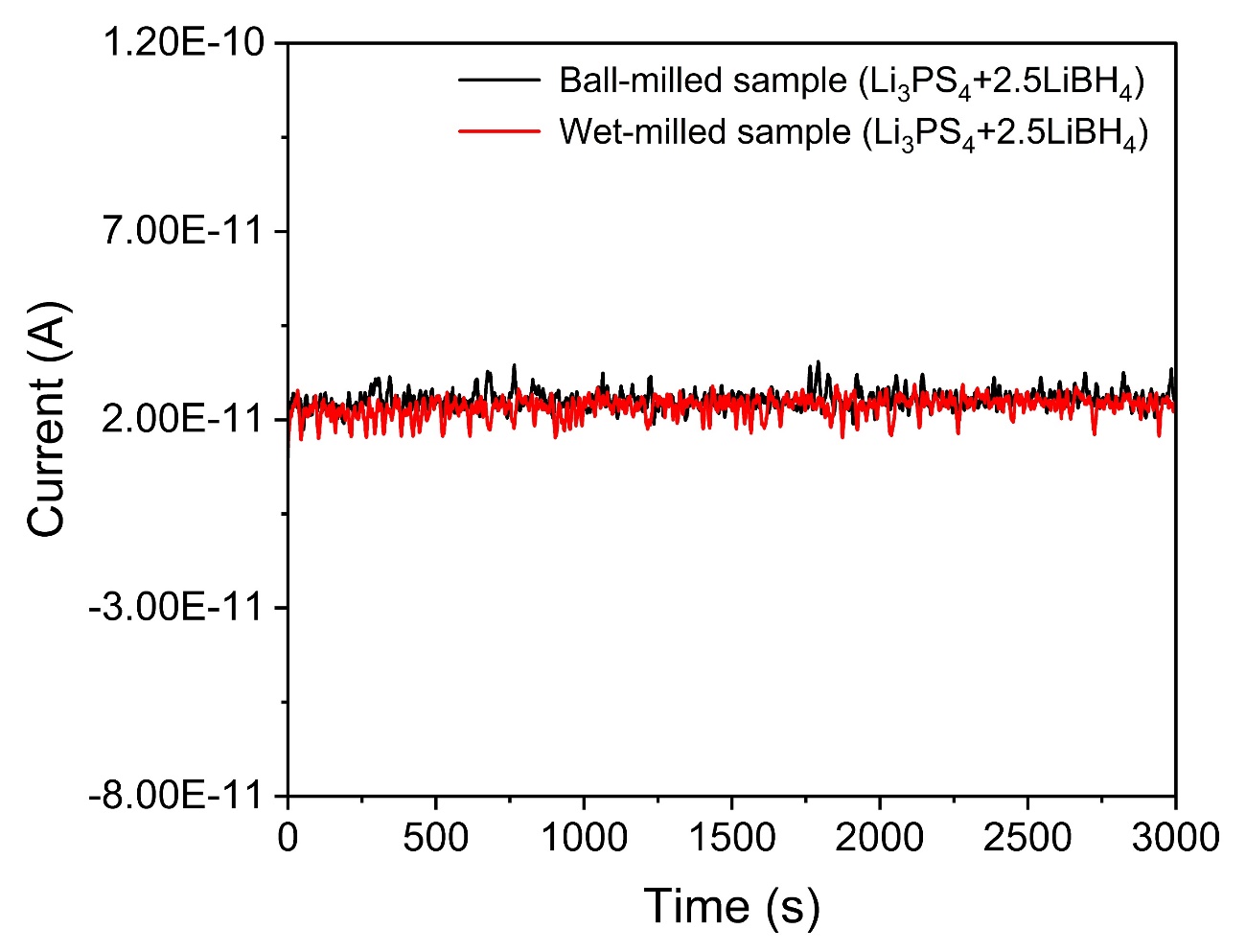
**Figure S12.** Direct current polarization curve of the synthesized samples. Chronoamperometry measurements were performed on samples synthesized by wet milling as well as by dry ball milling. The wet-milled sample was synthesized using 10mm balls and 13 cm^3^ of o-xylene as the solvent, followed by drying at 90 °C. The measurements were conducted while maintaining a constant voltage of 0.1 mV. The average current value for the wet-milled sample was 2.34×10^−11^ A, and for the ball-milled sample, it was 2.53×10^−11^ A. From these values, the electronic conductivity was calculated to be 9.10×10^−9^ S cm^−1^ for the dry ball-milled sample and 8.66×10^−9^ S cm^−1^ for the wet synthesized sample.

**Table S1.** Constraints employed for conducting Rietveld refinement on XRD data of the as-synthesized samples. The atomic positions of T5 were taken from Ref.^[1]^ and not refined in this study. H (B@4d) refers to hydrogen in BH_4_ with boron at the 4d site. Similarly, H (B@4a) refers to hydrogen in BH_4_ with boron at the 4a site.

| Atom | Site | x | y | z | Occupancy |
| --- | --- | --- | --- | --- | --- |
| Li | 48h (T5) | 0.3139 | 0.0219 | 0.6861 | (7-Occ1-Occ2)/12 |
| P | 4b | 0 | 0 | 0.5 | 1 |
| S | 16e | Pos1 | −Pos1 | 0.5+Pos1 | 1 |
| S | 4d | 0.25 | 0.25 | 0.75 | 1−Occ1 |
| B | 4d | 0.25 | 0.25 | 0.75 | Occ1 |
| S | 4a | 0 | 0 | 0 | 1−Occ2 |
| B | 4a | 0 | 0 | 0 | Occ2 |
| H (B@4d) | 16e | 0.31350 | 0.18650 | 0.81350 | Occ1/2 |
| H (B@4d) | 16e | 0.31350 | 0.31350 | 0.81350 | Occ1/2 |
| H (B@4a) | 16e | 0.06350 | −0.06350 | 0.06350 | Occ2/2 |
| H (B@4a) | 16e | 0.06350 | 0.06350 | 0.06350 | Occ2/2 |

**Table S2.** Rietveld refinement results of Li_3_PS_4_+2LiBH_4_ synthesized with 10 mm balls and 10 cm^3^ of o-xylene.

| Atom | Site | x | y | z | Occupancy |
| --- | --- | --- | --- | --- | --- |
| Li | 48h (T5) | 0.3139 | 0.0219 | 0.6861 | 0.46(4) |
| P | 4b | 0 | 0 | 0.5 | 1 |
| S | 16e | 0.1199(5) | −0.1199(5) | 0.6199(5) | 1 |
| S | 4d | 0.25 | 0.25 | 0.75 | 0.46(3) |
| B | 4d | 0.25 | 0.25 | 0.75 | 0.54(3) |
| S | 4a | 0 | 0 | 0 | 0.08(2) |
| B | 4a | 0 | 0 | 0 | 0.92(2) |
| H | 16e | 0.31350 | 0.18650 | 0.81350 | 0.268(13) |
| H | 16e | 0.31350 | 0.31350 | 0.81350 | 0.268(13) |
| H | 16e | 0.06350 | −0.06350 | 0.06350 | 0.462(12) |
| H | 16e | 0.06350 | 0.06350 | 0.06350 | 0.462(12) |
| Space group, F-43m; lattice parameter, a = 10.011(2) Å; Rwp = 3.635 | | | | | |

**Table S3.** Rietveld refinement results of Li_3_PS_4_+2.25LiBH_4_ synthesized with 10 mm balls and 10 cm^3^ of o-xylene (T5 site from Table S1).

| Atom | Site | x | y | z | Occupancy |
| --- | --- | --- | --- | --- | --- |
| Li | 48h (T5) | 0.3139 | 0.0219 | 0.6861 | 0.44(4) |
| P | 4b | 0 | 0 | 0.5 | 1 |
| S | 16e | 0.1201(5) | −0.1201(5) | 0.6201(5) | 1 |
| S | 4d | 0.25 | 0.25 | 0.75 | 0.33(3) |
| B | 4d | 0.25 | 0.25 | 0.75 | 0.67(3) |
| S | 4a | 0 | 0 | 0 | 0.00(2) |
| B | 4a | 0 | 0 | 0 | 1.00(2) |
| H | 16e | 0.31350 | 0.18650 | 0.81350 | 0.337(13) |
| H | 16e | 0.31350 | 0.31350 | 0.81350 | 0.337(13) |
| H | 16e | 0.06350 | −0.06350 | 0.06350 | 0.500(12) |
| H | 16e | 0.06350 | 0.06350 | 0.06350 | 0.500(12) |
| Space group, F-43m; lattice parameter, a = 10.015(2) Å; Rwp = 3.823 | | | | | |

**Table S4.** Rietveld refinement results of Li_3_PS_4_+2.5LiBH_4_ synthesized with 10 mm balls and 10 cm^3^ of o-xylene, dried at 90 °C (T5 site from Table S1).

| Atom | Site | x | y | z | Occupancy |
| --- | --- | --- | --- | --- | --- |
| Li | 48h (T5) | 0.3139 | 0.0219 | 0.6861 | 0.44(4) |
| P | 4b | 0 | 0 | 0.5 | 1 |
| S | 16e | 0.1197(5) | −0.1197(5) | 0.6197(5) | 1 |
| S | 4d | 0.25 | 0.25 | 0.75 | 0.25(3) |
| B | 4d | 0.25 | 0.25 | 0.75 | 0.75(3) |
| S | 4a | 0 | 0 | 0 | 0.00(3) |
| B | 4a | 0 | 0 | 0 | 1.00(3) |
| H | 16e | 0.31350 | 0.18650 | 0.81350 | 0.374(14) |
| H | 16e | 0.31350 | 0.31350 | 0.81350 | 0.374(14) |
| H | 16e | 0.06350 | −0.06350 | 0.06350 | 0.500(12) |
| H | 16e | 0.06350 | 0.06350 | 0.06350 | 0.500(12) |
| Space group, F-43m; lattice parameter, a = 10.0228(16) Å; Rwp = 4.021 | | | | | |

**Table S5.** Rietveld refinement results of Li_3_PS_4_+2.5LiBH_4_ synthesized with 10 mm balls and 7 cm^3^ of o-xylene, dried at 90 °C (T5 site from Table S1).

| Atom | Site | x | y | z | Occupancy |
| --- | --- | --- | --- | --- | --- |
| Li | 48h (T5) | 0.3139 | 0.0219 | 0.6861 | 0.44(4) |
| P | 4b | 0 | 0 | 0.5 | 1 |
| S | 16e | 0.1195(5) | −0.1195(5) | 0.6195(5) | 1 |
| S | 4d | 0.25 | 0.25 | 0.75 | 0.26(3) |
| B | 4d | 0.25 | 0.25 | 0.75 | 0.74(3) |
| S | 4a | 0 | 0 | 0 | 0.00(3) |
| B | 4a | 0 | 0 | 0 | 1.00(3) |
| H | 16e | 0.31350 | 0.18650 | 0.81350 | 0.372(14) |
| H | 16e | 0.31350 | 0.31350 | 0.81350 | 0.372(14) |
| H | 16e | 0.06350 | −0.06350 | 0.06350 | 0.500(13) |
| H | 16e | 0.06350 | 0.06350 | 0.06350 | 0.500(13) |
| Space group, F-43m; lattice parameter, a = 10.0274(15) Å; Rwp = 4.29 | | | | | |

**Table S6.** Rietveld refinement results of Li_3_PS_4_+2.5LiBH_4_ synthesized with 10 mm balls and 13 cm^3^ of o-xylene, dried at 90 °C (T5 site from Table S1).

| Atom | Site | x | y | z | Occupancy |
| --- | --- | --- | --- | --- | --- |
| Li | 48h (T5) | 0.3139 | 0.0219 | 0.6861 | 0.44(4) |
| P | 4b | 0 | 0 | 0.5 | 1 |
| S | 16e | 0.1184(5) | −0.1184(5) | 0.6184(5) | 1 |
| S | 4d | 0.25 | 0.25 | 0.75 | 0.26(3) |
| B | 4d | 0.25 | 0.25 | 0.75 | 0.74(3) |
| S | 4a | 0 | 0 | 0 | 0.00(3) |
| B | 4a | 0 | 0 | 0 | 1.00(3) |
| H | 16e | 0.31350 | 0.18650 | 0.81350 | 0.372(14) |
| H | 16e | 0.31350 | 0.31350 | 0.81350 | 0.372(14) |
| H | 16e | 0.06350 | −0.06350 | 0.06350 | 0.500(14) |
| H | 16e | 0.06350 | 0.06350 | 0.06350 | 0.500(14) |
| Space group, F-43m; lattice parameter, a = 10.0261(16) Å; Rwp = 4.42 | | | | | |

**Table S7.** Rietveld refinement results of Li_3_PS_4_+2.5LiBH_4_ synthesized with 10 mm balls and 10 cm^3^ of o-xylene, dried at 25 °C (T5 site from Table S1).

| Atom | Site | x | y | z | Occupancy |
| --- | --- | --- | --- | --- | --- |
| Li | 48h (T5) | 0.3139 | 0.0219 | 0.6861 | 0.44(4) |
| P | 4b | 0 | 0 | 0.5 | 1 |
| S | 16e | 0.1222(6) | −0.1222(6) | 0.6222(6) | 1 |
| S | 4d | 0.25 | 0.25 | 0.75 | 0.17(3) |
| B | 4d | 0.25 | 0.25 | 0.75 | 0.83(3) |
| S | 4a | 0 | 0 | 0 | 0.09(3) |
| B | 4a | 0 | 0 | 0 | 0.91(3) |
| H | 16e | 0.31350 | 0.18650 | 0.81350 | 0.414(14) |
| H | 16e | 0.31350 | 0.31350 | 0.81350 | 0.414(14) |
| H | 16e | 0.06350 | −0.06350 | 0.06350 | 0.454(14) |
| H | 16e | 0.06350 | 0.06350 | 0.06350 | 0.454(14) |
| Space group, F-43m; lattice parameter, a = 10.0203(17) Å; Rwp = 4.75 | | | | | |

**Table S8.** Rietveld refinement results of Li_3_PS_4_+2.5LiBH_4_ synthesized with 10 mm balls and 10 cm^3^ of o-xylene, dried at 50 °C (T5 site from Table S1).

| Atom | Site | x | y | z | Occupancy |
| --- | --- | --- | --- | --- | --- |
| Li | 48h (T5) | 0.3139 | 0.0219 | 0.6861 | 0.44(4) |
| P | 4b | 0 | 0 | 0.5 | 1 |
| S | 16e | 0.1217(6) | −0.1217(6) | 0.6217(6) | 1 |
| S | 4d | 0.25 | 0.25 | 0.75 | 0.25(3) |
| B | 4d | 0.25 | 0.25 | 0.75 | 0.75(3) |
| S | 4a | 0 | 0 | 0 | 0.00(3) |
| B | 4a | 0 | 0 | 0 | 1.00(3) |
| H | 16e | 0.31350 | 0.18650 | 0.81350 | 0.374(14) |
| H | 16e | 0.31350 | 0.31350 | 0.81350 | 0.374(14) |
| H | 16e | 0.06350 | −0.06350 | 0.06350 | 0.500(13) |
| H | 16e | 0.06350 | 0.06350 | 0.06350 | 0.500(13) |
| Space group, F-43m; lattice parameter, a = 10.0205(16) Å; Rwp = 4.12 | | | | | |

**Table S9.** Rietveld refinement results of Li_3_PS_4_+2.5LiBH_4_ synthesized with 10 mm balls and 10 cm^3^ of o-xylene, dried at 70 °C (T5 site from Table S1).

| Atom | Site | x | y | z | Occupancy |
| --- | --- | --- | --- | --- | --- |
| Li | 48h (T5) | 0.3139 | 0.0219 | 0.6861 | 0.44(4) |
| P | 4b | 0 | 0 | 0.5 | 1 |
| S | 16e | 0.1206(5) | −0.1206(5) | 0.6206(5) | 1 |
| S | 4d | 0.25 | 0.25 | 0.75 | 0.25(2) |
| B | 4d | 0.25 | 0.25 | 0.75 | 0.75(2) |
| S | 4a | 0 | 0 | 0 | 0.00(3) |
| B | 4a | 0 | 0 | 0 | 1.00(3) |
| H | 16e | 0.31350 | 0.18650 | 0.81350 | 0.374(12) |
| H | 16e | 0.31350 | 0.31350 | 0.81350 | 0.374(12) |
| H | 16e | 0.06350 | −0.06350 | 0.06350 | 0.500(13) |
| H | 16e | 0.06350 | 0.06350 | 0.06350 | 0.500(13) |
| Space group, F-43m; lattice parameter, a = 10.0207(13) Å; Rwp = 4.51 | | | | | |

**Table S10.** Rietveld refinement results of Li_3_PS_4_+2.5LiBH_4_ synthesized with 10 mm balls and 10 cm^3^ of o-xylene, dried at 90 °C (T5 site from Table S1).

| Atom | Site | x | y | z | Occupancy |
| --- | --- | --- | --- | --- | --- |
| Li | 48h (T5) | 0.3139 | 0.0219 | 0.6861 | 0.44(4) |
| P | 4b | 0 | 0 | 0.5 | 1 |
| S | 16e | 0.1199(6) | −0.1199(6) | 0.6199(6) | 1 |
| S | 4d | 0.25 | 0.25 | 0.75 | 0.25(3) |
| B | 4d | 0.25 | 0.25 | 0.75 | 0.75(3) |
| S | 4a | 0 | 0 | 0 | 0.00(3) |
| B | 4a | 0 | 0 | 0 | 1.00(3) |
| H | 16e | 0.31350 | 0.18650 | 0.81350 | 0.373(14) |
| H | 16e | 0.31350 | 0.31350 | 0.81350 | 0.373(14) |
| H | 16e | 0.06350 | −0.06350 | 0.06350 | 0.500(14) |
| H | 16e | 0.06350 | 0.06350 | 0.06350 | 0.500(14) |
| Space group, F-43m; lattice parameter, a = 10.0232(15) Å; Rwp = 5.57 | | | | | |

**Table S11.** Rietveld refinement results of Li_3_PS_4_+2.5LiBH_4_ synthesized with 10 mm balls and 10 cm^3^ of o-xylene, dried at 110 °C (T5 site from Table S1).

| Atom | Site | x | y | z | Occupancy |
| --- | --- | --- | --- | --- | --- |
| Li | 48h (T5) | 0.3139 | 0.0219 | 0.6861 | 0.46(3) |
| P | 4b | 0 | 0 | 0.5 | 1 |
| S | 16e | 0.1197(4) | −0.1197(4) | 0.6197(4) | 1 |
| S | 4d | 0.25 | 0.25 | 0.75 | 0.48(3) |
| B | 4d | 0.25 | 0.25 | 0.75 | 0.52(3) |
| S | 4a | 0 | 0 | 0 | 0.00(2) |
| B | 4a | 0 | 0 | 0 | 1.00(2) |
| H | 16e | 0.31350 | 0.18650 | 0.81350 | 0.262(13) |
| H | 16e | 0.31350 | 0.31350 | 0.81350 | 0.262(13) |
| H | 16e | 0.06350 | −0.06350 | 0.06350 | 0.500(12) |
| H | 16e | 0.06350 | 0.06350 | 0.06350 | 0.500(12) |
| Space group, F-43m; lattice parameter, a = 10.0227(10) Å; Rwp = 5.06 | | | | | |

**Table S12.** Rietveld refinement results of Li_3_PS_4_+2.5LiBH_4_ synthesized with 10 mm balls and 10 cm^3^ of o-xylene, dried at 130 °C (T5 site from Table S1).

| Atom | Site | x | y | z | Occupancy |
| --- | --- | --- | --- | --- | --- |
| Li | 48h (T5) | 0.3139 | 0.0219 | 0.6861 | 0.42(3) |
| P | 4b | 0 | 0 | 0.5 | 1 |
| S | 16e | 0.1187(4) | −0.1187(4) | 0.6187(4) | 1 |
| S | 4d | 0.25 | 0.25 | 0.75 | 0.64(2) |
| B | 4d | 0.25 | 0.25 | 0.75 | 0.36(2) |
| S | 4a | 0 | 0 | 0 | 0.00(2) |
| B | 4a | 0 | 0 | 0 | 1.00(2) |
| H | 16e | 0.31350 | 0.18650 | 0.81350 | 0.182(12) |
| H | 16e | 0.31350 | 0.31350 | 0.81350 | 0.182(12) |
| H | 16e | 0.06350 | −0.06350 | 0.06350 | 0.500(11) |
| H | 16e | 0.06350 | 0.06350 | 0.06350 | 0.500(11) |
| Space group, F-43m; lattice parameter, a = 10.0302(9) Å; Rwp = 4.85 | | | | | |

**Table S13.** Rietveld refinement results of Li_3_PS_4_+2.5LiBH_4_ synthesized with 10 mm balls and 10 cm^3^ of o-xylene, dried at 150 °C (T5 site from Table S1).

| Atom | Site | x | y | z | Occupancy |
| --- | --- | --- | --- | --- | --- |
| Li | 48h (T5) | 0.3139 | 0.0219 | 0.6861 | 0.45(3) |
| P | 4b | 0 | 0 | 0.5 | 1 |
| S | 16e | 0.1199(4) | −0.1199(4) | 0.6199(4) | 1 |
| S | 4d | 0.25 | 0.25 | 0.75 | 0.88(3) |
| B | 4d | 0.25 | 0.25 | 0.75 | 0.12(3) |
| S | 4a | 0 | 0 | 0 | 0.15(2) |
| B | 4a | 0 | 0 | 0 | 0.85(2) |
| H | 16e | 0.31350 | 0.18650 | 0.81350 | 0.061(13) |
| H | 16e | 0.31350 | 0.31350 | 0.81350 | 0.061(13) |
| H | 16e | 0.06350 | −0.06350 | 0.06350 | 0.423(10) |
| H | 16e | 0.06350 | 0.06350 | 0.06350 | 0.423(10) |
| Space group, F-43m; lattice parameter, a = 10.0253(9) Å; Rwp = 4.52 | | | | | |

**Table S14.** Rietveld refinement results of Li_3_PS_4_+2.5LiBH_4_ synthesized with 10 mm balls and 10 cm^3^ of o-xylene, dried at 200 °C (T5 site from Table S1).

| Atom | Site | x | y | z | Occupancy |
| --- | --- | --- | --- | --- | --- |
| Li | 48h (T5) | 0.3139 | 0.0219 | 0.6861 | 0.40(4) |
| P | 4b | 0 | 0 | 0.5 | 1 |
| S | 16e | 0.1237(6) | −0.1237(6) | 0.6237(6) | 1 |
| S | 4d | 0.25 | 0.25 | 0.75 | 1.00(4) |
| B | 4d | 0.25 | 0.25 | 0.75 | 0.00(4) |
| S | 4a | 0 | 0 | 0 | 0.74(3) |
| B | 4a | 0 | 0 | 0 | 0.26(3) |
| H | 16e | 0.31350 | 0.18650 | 0.81350 | 0.000(18) |
| H | 16e | 0.31350 | 0.31350 | 0.81350 | 0.000(18) |
| H | 16e | 0.06350 | −0.06350 | 0.06350 | 0.130(14) |
| H | 16e | 0.06350 | 0.06350 | 0.06350 | 0.130(14) |
| Space group, F-43m; lattice parameter, a = 9.9869(17) Å; Rwp = 3.97 | | | | | |

**References**

[1] A. Gautam, M. Sadowski, M. Ghidiu, N. Minafra, A. Senyshyn, K. Albe, W. G. Zeier, *Advanced Energy Materials* **2020**, *11* (5), https://doi.org/10.1002/aenm.202003369.
